# Multiple myeloma inhibitory effects of natural compounds: enhancement through nanoparticle carriers

**DOI:** 10.3389/fphar.2025.1589090

**Published:** 2025-06-17

**Authors:** Erica Wong, Anna Staskiewicz, Joshua Pruner, Jean Lee, Michael Tucker, Barkley Smith, Lanerica Rogers, Ganiat Asuni, Xinyu Wang

**Affiliations:** ^1^ Department of Inpatient Pharmacy, Lucile Packard Children’s Hospital Stanford, Palo Alto, CA, United States; ^2^ Graduate Division of Biological and Biomedical Sciences, Biochemistry, Cell and Developmental Biology Graduate Program, Emory University, Atlanta, GA, United States; ^3^ Doctor of Osteopathic Medicine Program, Philadelphia College of Osteopathic Medicine – Georgia Campus, Suwanee, GA, United States; ^4^ SSM Saint Louis University Hospital, St. Louis, MO, United States; ^5^ Doctor of Pharmacy Program, Philadelphia College of Osteopathic Medicine – Georgia Campus, Suwanee, GA, United States; ^6^ Department of Biomedical Sciences, Philadelphia College of Osteopathic Medicine – Georgia Campus, Suwanee, GA, United States

**Keywords:** multiple myeloma, nanoparticle carriers, 2-cyano-3,12-dioxooleana 1,9-dien-28-oic acid (CDDO), caffeic acid phenethyl ester (CAPE), xanthohumol (XN), resveratrol (RSV), curcumin (CUR), gallic acid (GA)

## Abstract

Natural compounds have emerged as promising therapeutic agents for treating cancers such as multiple myeloma (MM). However, poor bioavailability, low stability, and suboptimal targeting often limit their clinical efficacy. Recent advances in nanotechnology have addressed these limitations by utilizing nanoparticle (NP) carriers to enhance the therapeutic potential of natural compounds through improved solubility, stability, and selective delivery to cancer cells. This review explores the inhibitory effects of key natural compounds on MM cells, including 2-cyano-3,12-dioxooleana-1,9-dien-28-oic acid (CDDO) and its derivatives, caffeic acid phenethyl ester (CAPE) and its derivatives, xanthohumol (XN) and its derivatives, resveratrol (RSV) and its derivatives, curcumin (CUR), 3,4,5-trihydroxybenzoic acid (gallic acid; GA), and evodiamine (EVO). These compounds exhibit potent anti-proliferative, pro-apoptotic, and anti-inflammatory properties through the modulation of signaling pathways such as NF-κB, STAT3, and PI3K/Akt, which are critical in MM pathogenesis. Despite their therapeutic promise, the clinical application of these natural agents has been hampered by pharmacokinetic challenges. NP carriers, including liposomes, polymeric NPs, and lipid-based nanocarriers, have been engineered to improve these compounds’ bioavailability and targeted delivery, enhancing their cytotoxicity against MM cells. For instance, CDDO and its derivatives encapsulated in NPs have demonstrated increased intracellular accumulation and improved inhibition of NF-κB activity. Similarly, NP formulations of CAPE, XN, and RSV have enhanced anti-MM effects through improved stability and sustained drug release. CUR, known for its poor water solubility, has seen its therapeutic potential augmented through NP delivery systems, enabling higher drug concentrations at tumor sites. Though structurally distinct, GA and EVO have benefited from NP-based enhancement, exhibiting improved bioavailability and selective targeting of MM cells. This review highlights the promising role of NP carriers in overcoming the pharmacokinetic limitations of natural compounds, offering new avenues for more effective MM therapies.

## 1 Introduction

Multiple myeloma (MM) is a blood cancer characterized by the abnormal proliferation of plasma cells in the bone marrow and their production of monoclonal proteins (Kyle et al., 2003). MM is expected to be diagnosed in approximately 35,780 individuals in the US in 2024 (Siegel et al., 2024). The 5-year relative survival rate for MM is approximately 60% (Siegel et al., 2024), and the mortality rate within the first year of diagnosis ranges from 10 to 15 percent (Charliński et al., 2021). These poor survival outcomes for MM are a great concern despite continuous studies on the progression of new drug options in clinical practice. MM treatment options include the use of CAR-T cell therapy, monoclonal antibodies, steroids, proteasome inhibitors, and immunomodulatory drugs (Kumar et al., 2023).

Many compounds currently being studied involve natural compounds for their anti-inflammatory and apoptotic mechanism through various signaling pathways in different MM cell lines, such as RPMI 8226, U266, *etc.* Many of these naturally-derived compounds have shown prominent synergistic effects with another natural compound or with existing drug treatment options (Palliyage et al., 2021). These natural drug candidates are currently being studied as adjuvant or monotherapy on their therapeutic effects in various cancer disease states, including MM. Several known and/or novel candidates we cover in this review include 2-cyano-3,12-dioxooleana-1,9-dien-28-oic acid (CDDO) and its derivatives, caffeic acid phenethyl ester (CAPE) and its derivatives, xanthohumol (XN) and its derivatives, resveratrol (RSV) and its derivatives, curcumin (CUR), 3,4,5-trihydroxy benzoic acid (gallic acid; GA), and evodiamine (EVO).

The limitations of natural compounds as drug candidates include their low stability and bioavailability in the body, which can potentially hinder them as a therapeutic option (Kapetanovic et al., 2011). Recent studies examine the potential formulation of nanoparticle (NP) carriers to enhance the pharmacokinetic/pharmacodynamic profile of these compounds. NPs are colloidal drug carriers that refine drugs, proteins, and lipid layers into nanoscale formulations for cancer therapy (Swami et al., 2014). NP carriers display a more promising strategy for drug delivery to the bone marrow site, particularly when formulated with targeted ligands (Swami et al., 2014; Sun et al., 2023). Many current studies aim to examine the benefits of NPs as a novel cancer drug formulation. This review article aims to analyze the most relevant studies on a list of naturally derived compounds and NPs’ role in the delivery of these natural compounds in MM. We searched several databases, including PubMed, ScienceDirect, Scopus, and Google Scholar, using combinations of keywords for specific phytochemicals, NPs, and MM and limiting selected studies to those published by September 2024.

## 2 Multiple myeloma

### 2.1 Background of multiple myeloma

MM is a malignant cancer with a plasma cell neoplasm mainly located in the bone marrow microenvironment. In most MM cases, osteolytic bone lesions arise, albeit this is a slowly progressing form of cancer. Less than 1% of the total tumor cells in a patient with MM actively synthesize DNA until further disease progression (Kuehl and Bergsagel, 2002). MM is characterized by tumors that reside outside the bone marrow compartment, further circulating throughout the body. Multiple therapeutic strategies have been investigated, such as those that directly alter the tumor cells’ abilities regarding proliferation, differentiation, and apoptosis, which have achieved longer remission time and increased survival rates. However, the likelihood of relapse is a concern that, interestingly, plant-derived compounds may be more equipped to treat.

**FIGURE 1:**
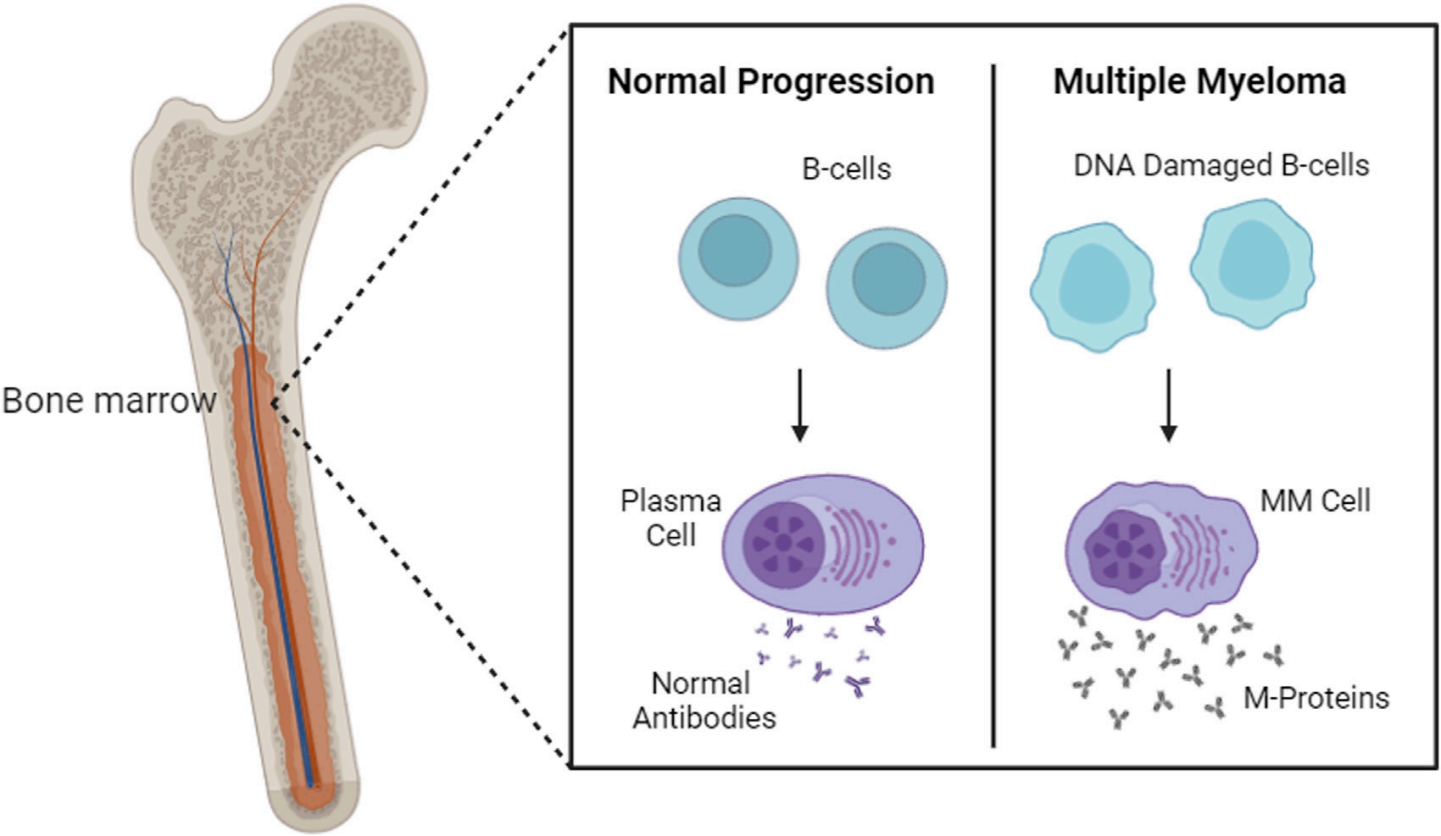
Healthy plasma cells *versus* proliferated MM cells. This figure was created through www.biorender.com (BioRender, Toronto, ON, Canada).

As malignant plasma cells grow and proliferate in the bone marrow, they start producing monoclonal proteins such as abnormal antibodies (e.g., IgG or IgM) or immunoglobulin light chains (e.g., Bence Jones proteins) (Figure 1). These abnormal antibodies and immunoglobulin chains infiltrate the bone marrow and cause extensive skeletal destruction and other characteristic findings. Normal bone marrow function is suppressed, leading to anemia, thrombocytopenia, and leukopenia, manifesting in fatigue, bleeding, and/or recurrent infections (Kyle et al., 2003). The overproduction of monoclonal immunoglobulin and light chains also increases serum viscosity and may rarely lead to hyperviscosity syndrome: syncope, headache, impaired vision, paranesthesia, and numbness (Mehta and Singhal, 2003). Clinical symptoms of MM include mild fever, night sweats, and weight loss. These abnormal proteins are also responsible for the different kidney manifestations in patients with MM, such as direct tubular toxicity, myeloma cast nephropathy, primary amyloidosis with renal involvement, and light chain deposition disease (Kyle et al., 2003).

The diagnostic workup for MM includes laboratory studies, urine studies, bone marrow biopsy, and radiologic evaluation (San-Miguel et al., 2013; Kumar et al., 2023). Radiologically, skeletal lesions are the hallmark of MM pathogenesis. On X-ray, MM lesions have a punched-out osteolytic appearance, but at least 50% of the involved trabecular bone needs to be destroyed to be detected with this technique. Computed tomography (CT) scans are much more sensitive than X-rays and can detect lesions with <5% of trabecular bone destruction. However, magnetic resonance imaging (MRI) is the most specific and sensitive test for detecting bone disease and soft tissue involvement in MM (Dimopoulos et al., 2015).

### 2.2 Mechanism of MM pathogenesis

The pathogenesis of MM is a complex process leading to the replication of a malignant plasma cell clone that originates from the lymphoid B-cell lineage and develops after lineage commitment in the bone marrow (Kazandjian et al., 2014). On a genetic basis, at the most basic level, MM involves a considerable amount of immunoglobulin-heavy chain hypermutations and chromosomal abnormalities, particularly in the form of primary and secondary translocations.

Multiple mechanisms for MM cell dysregulation have been described, such as translocations of the Cyclin D1 gene with the IgH gene, specific Cyclin D gene amplifications, trisomies, and other cytogenetic events (Bergsagel et al., 2005; Broyl et al., 2010). The primary translocation in MM usually involves the immunoglobulin heavy chain (IgH) gene locus on chromosome 14 (14q32) and one of several partner chromosomes, including chromosomes 4, 6, 11, 14, and 20 (Fonseca et al., 2003). The two most frequent translocations are 11q13, which directly targets and upregulates the Cyclin D1 gene, and 4p16, which targets both the FGFR3 and MMSET genes and leads to dysregulation of cyclin D2 (Fonseca et al., 2003). Both translocations result in a plasma cell clone producing a monoclonal immunoglobulin.

Dysregulation of cyclin D appears to be a necessary occurrence in the establishment of abnormal plasma cells. This may be mediated through events that include the loss of chromosome 13 (site of retinoblastoma tumor-suppressor gene) and the acquisition of mutations that lead to the activation of the MYC and RAS oncogenes (Chiecchio et al., 2009; Chng et al., 2011; Chng et al., 2008). Additional cytogenetic events that occur later in the course of the disease are associated with a poorer prognosis. For example, copy number changes of chromosome 1, loss of tumor suppressor TP53 activity, and mutations that activate nuclear factor kappa B (NF-κB) are all associated with late-stage disease of MM (Lodé et al., 2010; Wong et al., 2020).

The bone marrow stromal cell is a major bone marrow component and a significant constituent of the cellular element that can increase the proliferation and progression of MM cells (Bianco and Gehron Robey, 2000). The bone marrow stromal cell adhesion to MM cells activates pathways in MM cells that support tumor cell proliferation, migration, drug resistance, and the expression of anti-apoptotic proteins (Bianchi and Munshi, 2015). The most notable is the secretion of cytokine IL-6, which enhances the secretion of vascular endothelial growth factor (VEGF) by MM cells that support the growth of new blood vessels (Bianchi and Munshi, 2015).

When MM cells attach to bone marrow stromal cells, a cascade of signals occurs in many different pathways, which are still being elucidated. NF-κB, for instance, causes an increase in IL-6 (Manier et al., 2012). This pro-inflammatory cytokine induces the growth of tumor cells and the expression of anti-apoptotic factors, which has been implicated in multiple cancer studies (Altayli et al., 2015). More specifically, aberrant plasma cells exhibit an extraordinary ability to interact with bone marrow mesenchymal stem cells, osteoblasts, osteoclasts, endothelial cells, adipocytes, and the immune system to induce a state of immune suppression while promoting bone marrow remodeling to obtain the best conditions favoring aberrant plasma cell expansion and drug resistance (Pojero et al., 2019). The binding of MM cells to the stromal cells in the bone marrow microenvironment causes an increased number of cytokines and growth factors that further potentiate the progression of the disease (Marin et al., 2019).

### 2.3 Current treatment for MM

There is currently no known cure for MM, but in recent years, treatments, including chemotherapy and autologous hematopoietic cell transplantation (HCT), have dramatically improved the lives of patients with MM. Risk stratification helps determine the patient’s prognosis and treatment options (Rajkumar, 2019). A high-risk MM correlates with a more aggressive MM and a worse prognosis.

Standard treatment often involves triple therapy with chemotherapy treatment in combination with steroids and a proteasome inhibitor (Kumar et al., 2023). For example, initial treatment consists of a three-drug regimen, such as bortezomib (a proteasome inhibitor), lenalidomide, and dexamethasone (VRd) (Richardson et al., 2010). For those eligible for autologous HCT, four cycles of VRd are given, followed by stem cell collection (Richardson et al., 2010). Then, a decision is made regarding whether to proceed with autologous HCT or continue the same chemotherapy regimen, reserving HCT for the first relapse. For those ineligible for an HCT, 8–12 cycles of VRd are recommended, followed by bortezomib maintenance therapy. Toxicities of these drugs include thromboembolic events, peripheral neuropathy, cytopenia, fatigue, and gastrointestinal distress (Richardson et al., 2010). Many more drug regimens are now being created as several of these drugs are not well tolerated in patients with co-morbidities. For example, lenalidomide is teratogenic, and there are emerging concerns regarding an increased risk of primary and secondary malignancy (Niesvizky et al., 2007). Lenalidomide should also be avoided in patients with renal failure due to the increased propensity of causing severe neutropenia and is replaced by cyclophosphamide in some cases (Niesvizky et al., 2007). Patients, such as frail adults, may receive a two-drug regimen of lenalidomide plus dexamethasone (Benboubker et al., 2014). Newer studies and treatment recommendations, such as the MASTER trial, show supporting evidence for the combination treatment of daratumumab, carfilzomib, lenalidomide, and dexamethasone (Dara-KRd) for newly diagnosed MM patients (Costa et al., 2022). However, limitations and adverse drug reactions (ADRs) are observed with all MM treatments, as shown in Table 1.

**TABLE 1:** Standard MM treatment options, per 2024 NCCN Clinical Practice Guidelines (Kumar et al., 2023), and their limitations.

Category	Drug	ADRs/Side effects
Proteasome Inhibitor	Bortezomib Carfilzomib Ixazomib	Peripheral neuropathy, hematologic toxicities, fatigue, gastrointestinal toxicity, and cardiovascular complications. Long-term use often leads to drug resistance. (Merin and Kelly, 2014; Cole and Frishman, 2018)
Corticosteroid	Dexamethasone	Hyperglycemia, increased risk of infections, osteoporosis, insomnia, GI disturbances, mood swings, adrenal suppression. (Liu et al., 2013; Kenna et al., 2011)
Immunotherapy Drug	Lenalidomide Pomalidomide Thalidomide	Thrombocytopenia, neutropenia, thromboembolic events, fatigue, rash, peripheral neuropathy, increased risk of infections, teratogenic effects, and strict pregnancy prevention measures. (Palumbo et al., 2012; Ghobrial and Rajkumar, 2003)
Chemotherapy	Bendamustine Cisplatin Cyclophosphamide Doxorubicin Etoposide	Alopecia, myelosuppression, nausea, vomiting, cardiotoxicity (doxorubicin), mucositis, hemorrhagic cystitis (cyclophosphamide), nephrotoxicity (cisplatin), increases the risk of infections, and secondary malignancies. (Carvalho et al., 2014; Pabla and Dong, 2008; Matz and Hsieh, 2017; Friberg et al., 2002)
Car T-Cell Therapy	Idecabtagene vicleucel Ciltacabtagene autoleucel	Neurotoxicity (immune effector cell-associated neurotoxicity syndrome), hypogammaglobulinemia, increased risk of severe infections, cytokine release syndrome. (Neelapu et al., 2018; Cappell and Kochenderfer, 2023)
Monoclonal Antibodies	Daratumab Elotuxumab Isatuximab-irfc	Neutropenia, thrombocytopenia, infusion-related reactions, fatigue anemia, diarrhea, respiratory infections, herpes zoster reactivation (Shingles). (Chari et al., 2018; Lonial et al., 2015; Richardson et al., 2020)
Nuclear Export Inhibitor	Selinexor	Anorexia, nausea, vomiting, fatigue, thrombocytopenia, hyponatremia, diarrhea, anemia, leukopenia, increased risk of infections and weight loss. (Babar et al., 2024; Kastritis et al., 2023)

## 3 Anti-myeloma activity of plant-derived compounds

**FIGURE 2:**
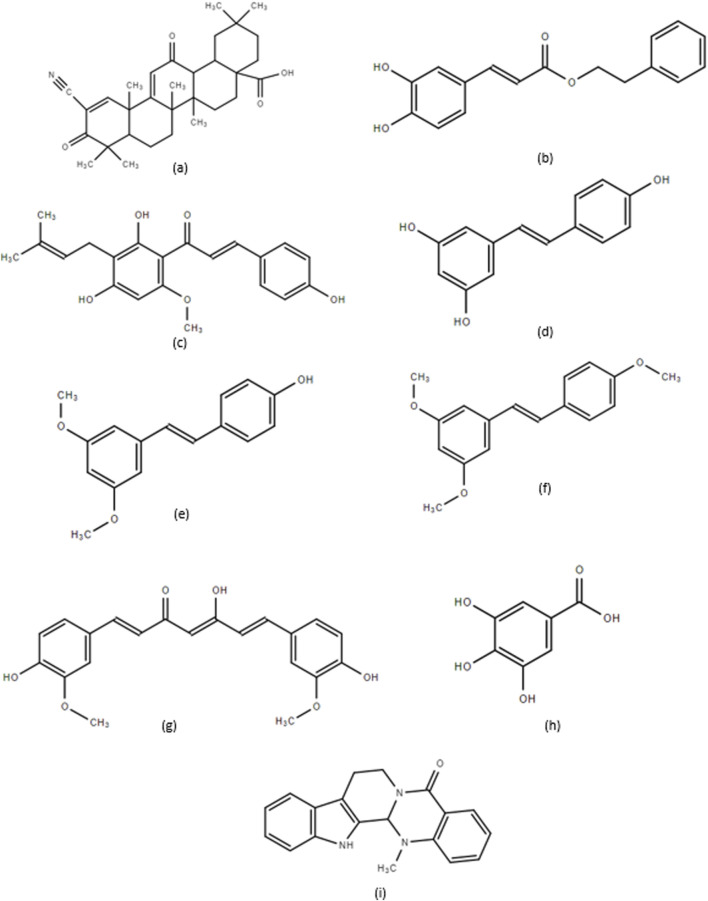
**(a)** 2-cyano-3,12-dioxooleana-1,9-dien-28-oic acid (CDDO), **(b)** Caffeic Acid Phenethyl Ester (CAPE), **(c)** Xanthohumol (XN), **(d)** Resveratrol (RSV), **(e)** Pterostilbene, **(f)** Trimethoxystilbene, **(g)** Curcumin (CUR), **(h)** Gallic Acid (GA), and **(i)** Evodiamine (EVO).

Natural compounds have constituted a large portion of new drugs developed within the past 4 decades (Newman and Cragg, 2020), and extensive research has shown that natural compounds display anticancer properties and the ability to overcome drug-induced resistance (Talib et al., 2021). This section reviews the action and mechanism of several natural compounds that inhibit MM cell growth. These compounds include CDDO and its derivatives, CAPE and its derivatives, XN and its derivatives, RSV and its derivatives, CUR, GA, and EVO, with their structures presented in Figure 2.

### 3.1 CDDO and derivatives

Many phytochemicals derived from plants have been shown to play a role in preventing and treating cancer. Triterpenoids are phytochemicals in foods containing oleanolic acids, such as olive oil and ursolic acids, and in fruits like apples and cranberries (Parikh et al., 2014). Due to the beneficial effects of these compounds, triterpenoids have been readily used in Asian countries for decades. Because oleanolic acid and ursolic acid are only weakly anti-tumorigenic *in vivo*, oleanane triterpenoid derivatives have been synthesized (Wang et al., 2014). Synthetic oleananes are more potent in blocking the production of inflammatory cytokines and chemokines from the tumor or immune cells (Liby and Sporn, 2012). These compounds are also potent activators of the Nrf2 pathway and can inhibit inducible nitric oxide synthase (iNOS) (Liby and Sporn, 2012).

Synthetic triterpenoid derivatives such CDDO are studied due to their antitumor activity. CDDO is potent at suppressing inflammatory enzymes that can lead to malignancy (Wang et al., 2014). To further increase the potency, various CDDO derivatives were synthesized to inhibit nitric oxide synthesis and function as antiproliferative agents for tumor cells (Petronelli et al., 2009). CDDO-methyl ester (ME) is one of the more potent derivatives that even made it to clinical trials involving pancreatic cancer, thyroid carcinoma, and mantle cell lymphoma patients (Wang et al., 2014). The promising findings with these synthetic triterpenoids have sparked the rise in studies addressing MM.

Research has shown that CDDO derivatives have a profound effect on MM. A study examining the anti-cancer effects of CDDO-methyl ester, CDDO-trifluoromethyl amide, and CDDO-imidazoline found that these CDDO derivatives decrease the viability of MM cells (Rogers et al., 2020). The proposed mechanism for the inhibitory effect on MM growth is through the induction of the intrinsic apoptotic pathway and the inhibition of cell cycle progression in the G0/G1 phase (Rogers et al., 2020). The inhibition of MM growth was additionally seen when co-culturing the cancer cells with human HS-5 stromal cells using a transwell model, further showing the compound’s potent effect even in conditions mimicking the bone marrow microenvironment (Rogers et al., 2020). This data supports CDDO and its derivatives in inhibiting MM cell growth.

LonP1 is a matrix protease with high expression linked to poor MM patient outcomes and resistance to proteasome-inhibiting treatments (Maneix et al., 2021). CDDO-Me has been shown to inhibit the function of LonP1 and, upon combinational treatment with proteasome inhibitor carfilzomib, increase the cytotoxic effects in MM cells (Maneix et al., 2021). The potential mechanism may involve the synergistic enhancement of protein stress in MM cells by inhibiting both proteasome and LonP1 (Maneix et al., 2021). The combination treatment of CDDO-Me with proteasome inhibitor bortezomib can have a similar mechanism of action (Maneix et al., 2021).

### 3.2 Caffeic acid phenethyl ester and derivatives

Polyphenols are naturally derived compounds found in various plants containing phenolic groups (Khushnud and Mousa, 2013). The two subsets of polyphenols include flavonoids and phenolic acids (Khushnud and Mousa, 2013). Due to polyphenols’ structure, they can act as antioxidants and show protective effects for plants and humans (Khushnud and Mousa, 2013). Due to the widespread knowledge of these natural compounds, research into similar therapeutic agents has been a significant field of study. Caffeic acid isopropenyl ester, caffeic acid benzyl ester, caffeic acid undecyl ester (CAUE), and CAPE are a few such compounds (Collins et al., 2019; Tomizawa et al., 2013). A study showed the cytotoxic effects of CAUE on NALM-6 cells, aggressive leukemia from an adolescent male, by increasing apoptosis in a concentration-dependent manner without harm to normal B lymphocytes (Tomizawa et al., 2013). CAPE is another specific phenolic acid found in honeybee resin (Collins et al., 2019). CAPE has been shown to have anti-microbial activity, anti-inflammatory activity, and anti-tumor effects (Tomizawa et al., 2013). The cytotoxic effects of CAPE were reported in various cancer cell lines, including human pancreatic cancer, ovarian cancer, C6 glioma cells, human colon cancer, and MM cells (Chen et al., 2008; Kleczka et al., 2020; Liu et al., 2018; Kuo et al., 2006; Xiang et al., 2006; Marin et al., 2019). CAPE has been shown to inhibit cell growth of 3 MM cell lines (RPMI 8226, NCI-H929, U266), while exhibiting no toxicity in normal peripheral blood B cells (Marin et al., 2019). Marin et al. observed that CAPE-treated MM cells had several upregulated oxidative stress response genes, such as heme oxygenase (decycling) 1, and apoptotic/DNA damage signaling genes, such as GADD45A and GADD45G (Marin et al., 2019).

The progression of MM is primarily supported by the altered bone marrow microenvironment (BMM). When MM cells attach to bone marrow stromal cells, a cascade of signals occurs (Manier et al., 2012). NF-κB regulates genes involved in cell proliferation (Cyclin D1), anti-apoptosis (e.g., survivin and the inhibitor of apoptosis protein), anti-cancer drug resistance (MDR1), cancer metastasis (e.g., COX-2 and MMP9), and immunomodulation (Takada et al., 2005). In a study on breast cancer cells treated with CAPE and several effective CAPE derivatives, these compounds showed activation of caspase-3 and caspase-7 apoptotic activity and similar potency of NF-κB modulation activity (Beauregard et al., 2015). These factors identify an anti-cancer mechanism of the upregulation of genes responsible for apoptotic factors and inhibition of NF-κB (Beauregard et al., 2015).

### 3.3 Xanthohumol and derivatives

XN is a prenylated flavonoid derived from *Humulus lupulus* L, a typical beer hop plant, and a member of the Cannabiaceae family utilized in the brewing process for its aromatic properties (Magalhães et al., 2009). Isolation of XN occurs from the lupulin glands of the hops plant, with the most numerous secondary metabolites sequestered at this site (Killeen et al., 2017). Previous studies have shown the potent pharmacological effects of XN, including antifungal, antiviral, antibacterial, anti-inflammatory, anti-obesity, antioxidant, and anti-cancer properties (Magalhães et al., 2009; Samuels et al., 2018).

Prenylated flavonoids are a group of biologically active, plant-derived polyphenols used for medicinal purposes for thousands of years and commonly found in vegetables, fruits, and tea (Kelly, 2010). The critical components of its structure include two phenolic rings and an adjoining prenyl group (Figure 2). In particular, the 5-carbon prenyl group confers lipophilic character to the molecule, increasing its bioavailability once ingested. XN has increasingly garnered interest from researchers due to the large quantities consumed annually through beer consumption worldwide. Although the concentration of 0.96 mg/L of XN found in a typical beer would not reach pharmacological dosages once ingested, the numerous health-promoting properties of XN warrant further study (Jiang et al., 2018).

The anti-myeloma effects of XN have recently begun to be investigated within the last few years. Sławińska-Brych et al. demonstrated that XN inhibits cell proliferation and induces apoptosis in MM cells through the production of reactive oxygen species (ROS), activation of the JNK and ERK pathways, and inhibition of VEGF production (Sławińska-Brych et al., 2019). Gallo et al. exhibited the XN-induced activation of adenosine monophosphate-activated protein kinase (AMPK), leading to the inhibition of endothelial cell function (Gallo et al., 2016), pointing to another possible mechanism through which XN exerts its effects. Tucker et al. confirmed XN’s apoptotic impact on MM cells through the extrinsic and intrinsic apoptotic pathways and the AMPK pathway’s involvement in this process (Tucker, 2020). Other non-myeloma anti-cancer activities of XN involve suppressing NF-κB-regulated gene products in leukemia cells by modifying p65 and IκBα kinase (Harikumar et al., 2009).

Under alkaline conditions and thermal treatment, XN is converted to its estrogenic form, isoxanthohumol (IXN). IXN is produced from XN during beer fermentation through wort boiling and is shown to be antiproliferative against breast, ovarian, prostate, and colon cancers (Magalhães et al., 2009; Żołnierczyk et al., 2015). Intestinal microbiota may activate up to 4 mg/L of IXN into another prenylated flavonoid, 8-prenylnaringenin (8-PN), a potent phytoestrogen with substantial inhibitory effects against breast and colon cancers (Possemiers et al., 2005; Koosha et al., 2019).

### 3.4 Resveratrol and derivatives

RSV (3,5,4′-trihydroxystilbene) is a naturally occurring cis- or trans-form polyphenol. Its methoxylated derivatives include pterostilbene (3,5-dimethoxy-4′-hydroxystilbene) and trimethoxystilbene (3,5,4′-trimethoxystilbene) (Schneider et al., 2003). The compounds can be found in grapes, berries, peanuts, and white wine (Nagarajan et al., 2022). RSV experiences a relatively quick first-pass metabolism, while the derivatives of RSV, such as pterostilbene and trimethoxystilbene, possess a longer half-life, slower elimination rate, and higher membrane permeability than its parent compound (Nagarajan et al., 2022; Mena et al., 2012). Pterostilbene contains two methoxy groups, resulting in increased lipophilicity and more resistance to phase I metabolism than the hydroxyl groups on RSV (Nagarajan et al., 2022). Trimethoxystilbene contains a third methyl group that may increase the molecule’s potency (Schneider et al., 2003).

RSV and its derivatives are known for their primary and secondary antioxidant properties (Haramizu et al., 2017). ROS are oxidative stress inducers introduced from a physiological stress response and are considered one of the underlying causes of cell death (Haramizu et al., 2017). RSV combats surplus oxidative stress and eventually reduces the mortality of normal cells in the body (Haramizu et al., 2017). Healthy viable cells in the bone marrow are essential to prevent further progression of the MM cells from replicating in a high-ROS environment. MM cells have increased ROS levels compared to normal cells and are more sensitive to treatments that induce oxidative stress (Caillot et al., 2021). Though RSV has antioxidative effects in normal cells, the compound has been shown to increase oxidative stress in MM cells treated with carfilzomib (Li et al., 2018). RSV derivative pinostilbene (3-methoxy-4′,5-dihydroxy-trans-stilbene) was reported to reduce the antioxidant expression in MM cells and increase apoptotic effects of bortezomib (Staskiewicz et al., 2023). Studies regarding combinatorial treatments of RSV and proteasome inhibitors illustrate the additive anti-MM potential of RSV treatment.

RSV has also shown autophagic and apoptotic effects in MM cells by mediating signaling pathways involving AMPK and the mammalian target of rapamycin (mTOR) (Ma et al., 2021). In 2018, Jin et al. observed that the adjunctive therapy of RSV with rapamycin targets malignant plasma cells by downregulating the mTOR signaling pathway (Jin et al., 2018). The results showed a significant reduction of cell proliferation among the MM cell lines through RSV and rapamycin combination therapy (Jin et al., 2018). Carfilzomib displays minimum modulation of stress-associated pathways in monotherapy, and through adjunctive therapy with RSV, both compounds synergistically reduce the mitochondrial protein SIRT1 stress-associated pathway (Li et al., 2018). Expression of both treatments shows downregulation of MM survival rate by blocking autophagy in MM cells (Li et al., 2018). The results of Li’s studies further expand on the disruption of mitochondrial proteins to increase the apoptotic stress on MM cells and maintain a balance of antioxidant activity in normal, non-MM cells (Haramizu et al., 2017; Li et al., 2018). Multitherapy options with RSV and its derivatives offer a higher efficacy and potency in apoptotic mechanisms in MM cell treatments (Jin et al., 2018; Li et al., 2018).

Pterostilbene is a dimethylated derivative of RSV with greater bioavailability (Kapetanovic et al., 2011). In 2012, Mena et al. found that pterostilbene caused cancer cell death by triggering lysosomal membrane permeabilization (Mena et al., 2012). Pterostilbene may offer a similar mechanism in MM cells. Other anti-MM mechanisms include apoptosis induction and cell cycle arrest in the G0 and G1 phases (Xie et al., 2016). Pterostilbene has also been shown to induce apoptosis in MM cells by activating AMPK (Mei et al., 2018). The compound DCZ0801 is a combination of pterostilbene and osalmide that was shown to reduce MM proliferation through the inhibition of glycolysis (Feng et al., 2020). In addition, Traversi et al. observed that the potent RSV derivative, 3,5,4′-trimethoxystilbene, disrupts tubulin polymerization in HeLa human cervical cancer cells (Traversi et al., 2017). In 2019, the same group of researchers found that a novel derivative 3,4,4′-trimethoxylstilbene was more effective than 3,5,4′-trimethoxystilbene in inducing mitotic arrest of HCT116 human colon cancer cells by inhibiting γ-tubulin (Traversi et al., 2019). Microtubule targeting compounds have shown efficacy in targeting MM cells (Rozic et al., 2016), indicating trimethoxystilbene could inhibit the proliferation of rapidly dividing cells like MM by disrupting y-tubulin and, consequently, centrosomal activity. In future studies, trimethoxystilbene can be examined as a potential or mono-adjunctive drug therapy for its mechanism in MM cells.

### 3.5 Curcumin

CUR is a significant component of turmeric, or *Curcuma longa*, which is classified into the Zingiberaceae ginger family (Santosa et al., 2022). CUR has been shown to inhibit MM cell growth *via* the inhibition of STAT3 phosphorylation (Bharti et al., 2003). A phase I/II study observed the downregulation of STAT3, NF-κB and COX-2 in MM patients treated with CUR (Vadhan-Raj et al., 2007). CUR possesses potent anti-inflammatory effects, targeting the NF-κB pathway in several cancers (Santosa et al., 2022). A randomized controlled trial in 2022 supports CUR’s anti-inflammatory mechanism with a significant remission rate of 75% compared to 33% in the melphalan/prednisone/CUR (MPC) group and melphalan/prednisone (MP) group, respectively (Santosa et al., 2022). The MPC-treated patients had decreased levels of NF-κB, tumor necrosis factor-alpha (TNF-α), and VEGF (Santosa et al., 2022). CUR was also found to sensitize MM cells to bortezomib treatment by regulating NF-κB, possibly through the JNK pathway (Bai and Zhang, 2012). CUR has been studied in several additional clinical trials, indicating the tolerability of CUR and alleviation of MM markers in patients with monoclonal gammopathy of undetermined significance and smoldering MM (Golombick et al., 2009; Golombick et al., 2012). Because of its safety profile, CUR has great potential as an anti-MM treatment.

One significant pathway thoroughly studied is CUR’s regulation of BRCA gene expression. CUR has sensitized melphalan-treated multidrug-resistant MOLP-2/R MM cells by further inhibiting the FA/BRCA pathway (Xiao et al., 2010). There is a linkage between the expression of specific gene pathways, such as FANCD2, to the FA/BRCA signaling pathway to inhibit the G2 MM cell growth phase (Xiao et al., 2010). The BRCA pathway is a signaling pathway that helps to induce growth, repair, and maintain healthy, normal cells (Chen et al., 2017). Chen et al. observed that CUR increased the resistance of normal bone marrow cells to carboplatin, an anti-tumor agent whose dose is limited due to side effects such as myelosuppression (Chen et al., 2017). CUR upregulated BRCA1 and BRCA2 expression, reducing the DNA damage induced by carboplatin in normal cells (Chen et al., 2017).

The CUR mechanism of apoptosis is evident in cancer-associated fibroblasts through the increased intracellular ROS production in the targeted cancer site (Zeng et al., 2020). Cancer-associated fibroblasts (CAFs) are the main factors in predicting malignant progression (Zeng et al., 2020). Studies associated with bone metastasis include the role of fibroblasts in inducing the motility and collagen production of cancer cell lines (Giannoni et al., 2010). Zeng et al. observed CUR’s inhibitory activity in prostate-CAFs (prostate cancer cell line) cells after 24-h treatment (Zeng et al., 2020). The ROS levels of prostate-CAFs contribute to an increase in cell membrane stress to induce apoptosis (Zeng et al., 2020). Similar reasoning can be introduced to MM cells and CUR treatment. Allegra et al. reported that CUR treatment increases ROS production in MM U266 cells (Allegra et al., 2018). Through the combination treatment of carfilzomib with CUR, significant cytotoxic apoptosis and cell cycle arrest were evident in the G1 phase (Allegra et al., 2018). One proposed mechanism of induced apoptosis and slow cell growth is the action of carfilzomib with CUR to suppress the NF-κB pathway of the MM cell cycle (Allegra et al., 2018). The targeting mechanisms of CUR in MM can be further examined with the suppressor gene expression p53 and other signaling pathways, like BRCA (Xiao et al., 2010; Allegra et al., 2018; Chen et al., 2017).

Using a combination of CUR and arsenic trioxide, Han et al. observed that CUR increased the cytotoxicity of MM U266 cell lines by increasing arsenic uptake in those cells with p53 mutations (Han et al., 2021). The combined treatment of both drugs also offers a potential treatment option for relapsing MM patients (Han et al., 2021). Although CUR has been reported to be safe at high doses (12g/day), the bioavailability of this compound is still low (Dei Cas and Ghidoni, 2019).

### 3.6 Gallic acid

GA, known by its chemical name 3,4,5-trihydroxy benzoic acid, comes from various natural sources, including bananas, strawberries, lemons, red wine, and green tea (Asci et al., 2017). Regarding its structure (Figure 2), GA has three hydroxyl groups attached to an aromatic ring in an ortho position, which explicitly provides its strong ability to scavenge ROS (Elwakeel and Abdel Rahman, 2021). However, a significant drawback to the therapeutic application, like many other plant-derived compounds, is the low bioavailability when given orally; moreover, GA absorption within the body is fast, but its maximum drug concentration in the plasma is very low (de Cristo Soares Alves et al., 2016). GA has been shown to have apoptotic and anti-angiogenic effects across several cancer types (Verma et al., 2013), which makes this compound particularly attractive to elucidate its mechanisms further.

GA’s notable qualities in terms of its cytoprotective effects are that it is a potent chelating agent and protects human cells or tissues from oxidative stress owing to its antioxidant, anti-inflammatory, and anticancer properties (Vijaya Padma et al., 2011). More specifically, GA has been shown to enhance regeneration and repair of the liver and kidneys as well as preserve the cells’ plasma membrane integrity (Vijaya Padma et al., 2011). Another study involving GA has shown that the compound improved the antioxidant status of cyclophosphamide-treated male albino rats through increasing glutathione levels, likely due to its theorized ability to elevate levels of antioxidant enzymes (Elwakeel and Abdel Rahman, 2021). A study on human vascular endothelial cells showed that GA rescued cells from cell death induced by homocysteine, adenosine, and TNF treatment (Kam et al., 2014). GA restored depleted DNA methyltransferase one and inhibited the proteasome (Kam et al., 2014). This suggests that GA is a potential candidate for inhibiting the 20S proteasome, further underlining its potential role in inhibiting MM cell proliferation. Kim et al. observed that GA and its derivatives, tannic acid and epigallocatechin-3-gallate, exhibited cytotoxic effects on MM cell lines RPMI 8226 and U266 (Kim et al., 2009). However, upon combination treatment with the proteasome inhibitor bortezomib, adding polyphenols partially inhibited the anti-MM effect of bortezomib by inactivating the boronic acid component (Kim et al., 2009). This highlights the importance of researching drug interactions, particularly when identifying potential synergistic combinations of natural compounds with other drugs.

With the lack of *in vivo* data, the anti-MM effect of GA has not been studied in clinical trials at the time of this review. However, a phase II study did show that daily consumption of pomegranate juice (570 mg of GA equivalents) slowed the progression of prostate-specific antigen in men who received surgery or radiation for prostate cancer (Pantuck et al., 2006). Epigallocatechin gallate is currently being assessed for potential effects on reducing liver cancer risk in patients with liver cirrhosis (Hoshida, 2023).

### 3.7 Evodiamine

EVO is an indole alkaloid extracted from the dried, unripe fruits of evodia (Evodiae rutaecarpa Bentham of the family Rutaceae). It is the principal biologically active constituent of unripe evodia fruit (Tan and Zhang, 2016). EVO elicits various pharmacological effects *via* various mechanisms, including anti-obesity, anti-bacterial, anti-viral, analgesic, anti-inflammatory, and anti-cancer effects (Tan and Zhang, 2016). The anti-cancer effects of EVO depend on the type of tumor being targeted. Various studies have demonstrated that EVO exhibits anti-tumor effects against MM by inducing pro-apoptotic and cell cycle arrest pathways (Fang et al., 2019). The pro-apoptotic effects of EVO are due to its suppressive effects on NF-κB, which is an important transcription factor in tumor development. Carcinogens such as cigarette smoke have been demonstrated to activate NF-κB, and the ability of EVO to inhibit the pro-carcinogenic downstream effects of NF-κB activation is responsible for its pro-apoptotic and anti-metastatic effects (Takada et al., 2005). In addition, EVO has been demonstrated to enhance the anti-cancer effects of the proteasome inhibitor, bortezomib, in MM cells, and this may be due to its inhibitory effect on MDR1 (Fang et al., 2019).

EVO exerts beneficial effects on cancer *via* different mechanisms. One proposed mechanism for its anticancer effect is its agonistic effects on transient receptor potential vanilloid 1 (TRPV1). EVO has been shown to induce ROS-dependent cytotoxicity and apoptotic cell death in human gastric cancer cells (Ivanova and Spiteller, 2014; Liu et al., 2022a). Also, TRPV1 activation induces Ca^2+^ influx, which is important for EVO-induced cytotoxicity through Ca^2+^ overload and endoplasmic reticulum stress culminating in cell death (Liu et al., 2022a). Another suggested mechanism is its antagonistic effects on the aryl hydrocarbon receptor (AhR), which modulates the expression of oncogenes, resulting in its anticancer effect (Yu et al., 2010). Furthermore, EVO has been shown to have antitumor activity against drug-resistant breast cancer *via* its anti-proliferative activity due to microtubule polymerization and apoptotic activity (Liao et al., 2005). The anticancer effect of EVO in human colon cancer has also been demonstrated, an effect that is partly attributed to its downregulation of Hypoxia-inducible factor 1-alpha (HIF-1α) in cancer cells through inhibition of IGF-1/PI3K/Akt signaling (Huang et al., 2015). Of the natural compounds covered in this study, EVO has been studied the least in its application to MM specifically. Additional anti-MM research is suggested, considering the efficacy and mechanistic insights in other cancers.

### 3.8 Summary of the mechanisms of anti-myeloma action

**FIGURE 3:**
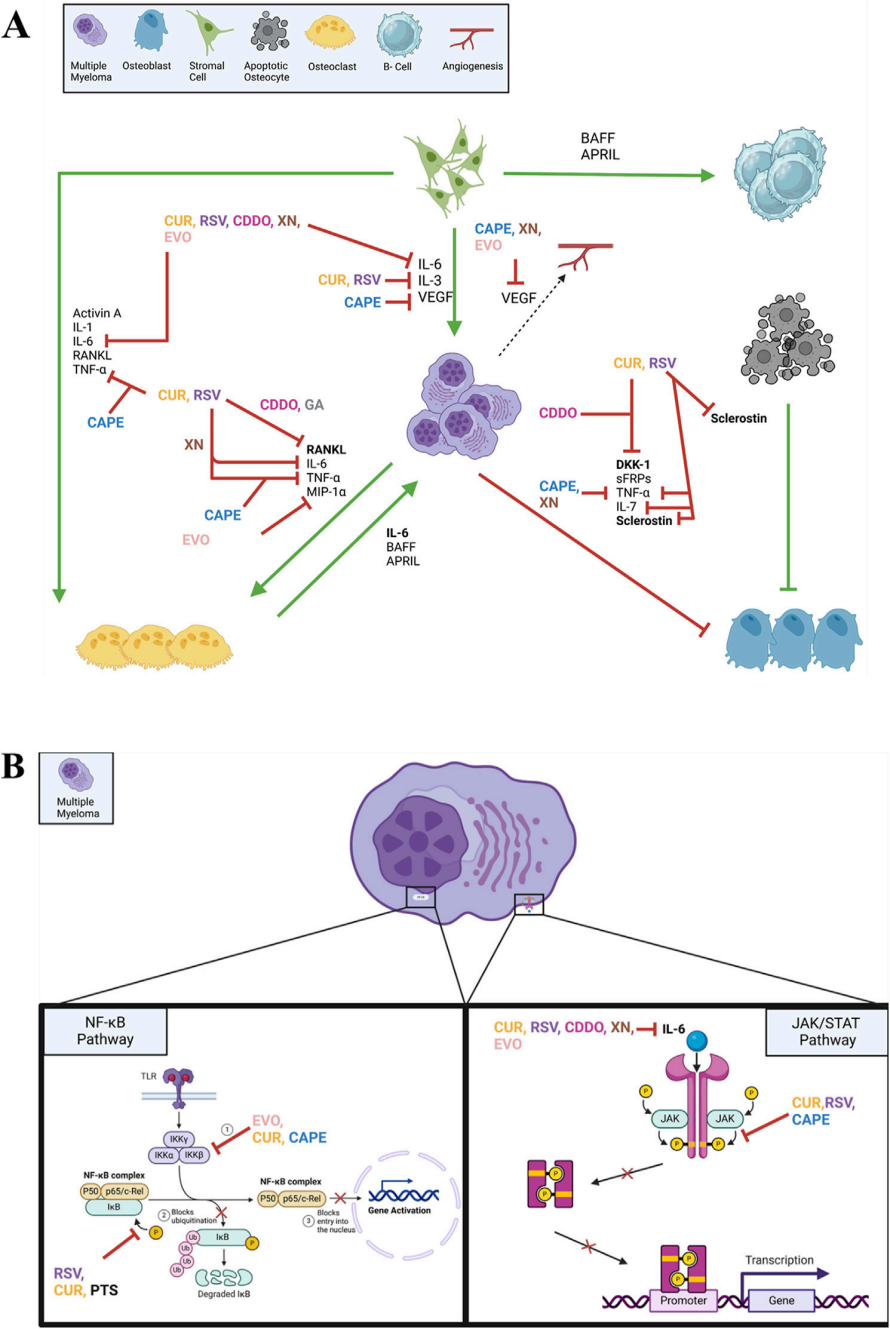
Mechanism of anti-myeloma action of natural compounds, including CAPE, CUR, RSV, PTS, COOD, XN, EVO, by targeting MM microenvironment **(A)** and NF-kB and JAK/STAT signaling pathways within MM cells **(B)**. This figure was created through www.biorender.com (BioRender, Toronto, ON, Canada).

These natural compounds demonstrate remarkable anti-MM effects through multiple interconnected molecular mechanisms. The compounds primarily modulate five key pathways: cell cycle regulation, apoptosis induction, NF-κB signaling, JAK/STAT signaling, and oxidative stress management. Through cell cycle regulation, they induce G1/S and G2/M arrest while downregulating cyclins and CDKs. The compounds trigger both intrinsic and extrinsic apoptotic pathways, leading to caspase activation and subsequent cell death. A crucial shared mechanism is the inhibition of the NF-κB pathway, achieved through suppression of IκB phosphorylation and prevention of p65 nuclear translocation, thereby reducing the expression of anti-apoptotic proteins. Notably, these compounds effectively suppress the JAK/STAT signaling pathway, particularly through inhibition of JAK2 and STAT3 phosphorylation, which plays a critical role in myeloma cell proliferation and survival. Additionally, these compounds modulate cellular redox status, either through ROS generation (as seen with CAPE and EVO) or through antioxidant effects (demonstrated by RSV and CUR). The stilbene derivatives (RSV, pterostilbene, and trimethoxystilbene) share structural similarities that contribute to their comparable molecular targets, while compounds like CDDO and curcumin exhibit particularly potent effects through simultaneous targeting of multiple pathways. This multi-targeted approach explains their potent anti-myeloma effects and suggests their potential as therapeutic agents or adjuvants in MM treatment. Their proposed mechanisms of action on MM are illustrated in Figure 3.

## 4 Application of nanoparticles to enhance the anti-cancer action of natural compounds

Nanotechnology has emerged as a promising strategy to enhance the therapeutic potential of natural compounds in cancer treatment (Sell et al., 2023). The natural compounds discussed previously, although they possess significant anti-cancer properties, often have limited clinical application due to poor bioavailability, rapid metabolism, and low solubility in the aqueous phase (Andreani et al., 2024). NPs are tiny particles with sizes typically ranging from 1 to 100 nm. The incorporation of these NPs provides a solution to overcome the challenges that natural compounds face by improving drug delivery, enhancing cellular uptake, and ensuring targeted release at tumor sites (Andreani et al., 2024). NP-based drug delivery systems, such as liposomes, polymeric NPs, and metal-based NPs, provide controlled release mechanisms that increase the stability and bioactivity of natural compounds while minimizing off-target effects. In this section, we focused on the targeted delivery and enhanced anti-MM effects of those selected natural compounds through the application of various forms of NPs.

### 4.1 CDDO and derivatives

CDDO has been shown to have anti-growth effects on several cancers, including MM. One main obstacle in using these compounds is how they are delivered to the target site. Many compounds undergo a first-pass effect, which can reduce the drug’s potency. The use of CDDO and its derivatives through NPs is a relatively new concept, as only a few published works on the topic exist. While no data shows CDDO delivery through NPs in treating MM, other research shows how CDDO derivatives can be used in treating other cancers through targeted NPs. The delivery of CDDO-Me through NPs has been shown to enhance the therapy against melanoma. Zhao et al. encapsulated CDDO-Me into a poly (lactic-co-glycolic acid) PLGA NP to deliver this compound to melanoma tumor-associated immune cells (Zhao et al., 2015). Intravenous delivery of CDDO-Me using NPs along with a subcutaneous vaccine increased the anti-cancer effect compared with injecting the vaccine alone (Zhao et al., 2015). CDDO delivery through the NP system has also been recently studied in various disease states to support the relief of inflammatory states, including atherosclerotic plaque build-up and lung cancer (Maiocchi et al., 2022; Guo et al., 2022). CDDO-loaded NPs may have potential for future studies in regards to treating MM cells.

### 4.2 CAPE

Research has gone into the effects of loading CAPE into NPs to increase its bioavailability. A study by Tambuwala et al. incorporated CAPE and piceatannol (PIC), another polyphenol found in grapes and wine, into albumin NPs to determine how well they work as therapeutic agents in C57BL mice with experimental colitis (Tambuwala et al., 2019). Like cancer, colitis is characterized by an upregulation in both hypoxia-inducible factor (HIF-1α) and NF-κB (Tambuwala et al., 2019). Following immunohistochemistry observations, HIF-1α and p65 expression, a subunit involved in the formation of NF-κB, were both decreased in the PIC/CAPE-loaded albumin NPs compared to control and freely administered PIC/CAPE (Tambuwala et al., 2019). This emphasizes the potential of studying combinatorial natural compounds that are anti-MM and increasing their bioavailability and targeting *via* NP carriers.

In another study, CAPE was encapsulated in a polyethylene glycol (PEG) coated polymer with anti-mortalin antibodies that were used for targeting and then examined uptake in various cancer cell lines (Wang et al., 2020). The cancer cell lines that showed the most uptake were those with high levels of mortalin on their surface (Wang et al., 2020). Compared to CAPE-PEG and CAPE alone, the CAPE-PEG with anti-mortalin antibodies decreased cell viability in a dose and time-dependent manner in lung carcinoma A549 cells (Wang et al., 2020). This evidence points to the potentially significant effects targeting NPs can have. Recently, our research group encapsulated CAPE onto iron oxide NPs (IONPs) conjugated with an RGD ligand targeting αvβ3 integrin, achieving targeted delivery to MM cells with improved stability and controlled drug release in acidic tumor environments (Smith et al., 2024). The formulated RGD-IONP/CAPE exhibited enhanced cytotoxicity and apoptosis induction in MM cells while sparing normal cells, suggesting its potential as a targeted therapeutic strategy for MM treatment​.

### 4.3 Xanthohumol and derivatives

It has been previously demonstrated that XN may be conjugated to ultra-small superparamagnetic iron oxide NPs (Khaki Najafabadi et al., 2017). Once within the body, these particles loaded with XN may be directed to a specific site of action under an external magnetic field.

Loading XN onto an electrospun poly (lactide-*co*-glycolide) (PLGA) fibrous mesh through an electrospinning technique has been employed with therapeutic benefit (Qiao et al., 2016). This process can use most polymers to produce continuous nanofibers for constructing a mesh-like network. Electrospun fibrous mesh medicated with XN has a high surface area to volume ratio, improved therapeutic effects, and little-to-no cytotoxicity on healthy cells (Qiao et al., 2016). Another study on the effect of Xn-loaded PLGA NPs on melanoma shows a statistically significant difference and improvement in XN-induced polarization with anti-inflammatory activities in CD86, a type 1 membrane immunoglobulin expressed in B cells and macrophages (Fonseca et al., 2021). Further outcomes display the gradual stability of XN-loaded NPs after freeze drying, achieving a significant decrease of approximately 70% in tumor melanocyte viability (Fonseca et al., 2021).

Different formulations of NP components were studied on general effects against plasma cancer cells that offer the potential formulation for MM cells as well (Harish et al., 2022). Optimized XN-loaded solid lipid NPs, prepared through homogenization and ultrasonication, display an improvement in XN’s poor bioavailability (Harish et al., 2022). Hydroxyapatite (HA) has also been blended into these mesh-like fibers as bone tissue scaffolds to improve biological functionality. HA’s unique mesoporous rod structure further improves the absorption of various chemicals and thus enhances anti-cancer therapy (Richard et al., 2021). The mesoporous rod structure shows adequate stabilization and remarkable inhibition of cancer cell growth, particularly studied with gambogic acid (Liu et al., 2022b). This feature allows for HA to be utilized as a drug carrier for a compound like XN. The use of an HA carrier is particularly interesting in regard to MM, which primarily localizes to the bone marrow and causes osteolytic bone lesions. HA nanoparticles have been reported to reduce bone tumor size *in vivo* and increase bone regeneration (Zhang et al., 2019). Considering the localization of MM cells, Qi et al. developed a bortezomib-carrying NP with alendronate, which binds to calcium phosphate in bones, and a MM cell membrane coat (Qi et al., 2024). MM mice treated with this NP (T-PB@M) had the best survival rate and experienced the greatest decrease in MM-associated symptoms compared to NP formulations without alendronate (Qi et al., 2024). Anti-MM NP design has the potential to harness specific targeting towards the bone in addition to MM cells themselves, as further discussed in the following section.

### 4.4 Resveratrol and derivatives

Nanotechnology provides a more applicable dosage delivery to reduce the likelihood of RSV from undergoing fast metabolism before reaching the bone marrow target site. A study used an HPLC instrument to examine the application of RSV, compared to a modified RSV structure (Singh et al., 2014). Catechin, a modified polyphenol that shares structural similarities to RSV, is a better and more stable drug candidate to be incorporated into RSV nanocarrier systems. The results show that the compound catechin has a higher retention rate and a stronger resistance to degradation in plasma samples (Singh et al., 2014). Other applications to show efficacy in delivering RSV and its derivatives include the process of encapsulation (Liu et al., 2020).

NPs made with casein, a food-grade protein, prevent RSV from being inactivated to the cis-isomerization (Peñalva et al., 2018). Casein NP carrier system increased the oral bioavailability of RSV tenfold by non-specifically binding to RSV and enhancing the protection in oral delivery (Peñalva et al., 2018). The results of casein as nanocarriers offer a more controlled release rate of the active trans-RSV. Thus, casein NPs increase the chances of RSV being absorbed into the plasma (Peñalva et al., 2018). The conjugated compound of zein/fucoidan (FU) offers a natural drug-delivery carrier for the RSV derivative pterostilbene, with no toxic effects seen in tested normal cells (Liu et al., 2020). The process of evaluating different NP carriers is important to maximize the efficacy of the loaded compound. In this study, the stability of the NPs varied based on the mass ratio of zein to FU, with the 2:1 NP exhibiting the greatest stability, and the release of pterostilbene was slower in pterostilbene-zein/FU (2:1) NPs compared to pterostilbene-zein NPs (Liu et al., 2020). In simulated digestion *in vitro*, pterostilbene-zein/FU displayed a sustained release of pterostilbene for up to 6 h in intestinal fluid (Liu et al., 2020). Incidents of rapid release were also present in Liu’s 2020 studies (Liu et al., 2020). Even so, the sustainability of NPs shows evidence of a more stabilized dosage form through the oral route, compared to the natural compound alone (Peñalva et al., 2018; Liu et al., 2020).

Intravenous injection provides 100% bioavailability into the body that allows a bypass of liver metabolism, enhancing the drug compound’s therapeutic efficacy. Wu et al. examined the tissue distribution of glycyrrhizic acid-conjugated human serum albumin-loaded RSV NP (GL-HSA-RESNP) in rats through tail vein injection (Wu et al., 2020). The results showed that for this treatment, compared to the injection of RSV alone, the *C*_max_ in the heart, liver and other organs was greater, the T_*max*_ was shorter, and the bioavailability was increased (Wu et al., 2020). The excretion phase in rats was examined within 4–24 h after administration of both GL-HSA-RESNPs and RSV alone. The RSV NP system displays a lower chance of toxicity in the rats’ organ systems (Wu et al., 2020). Similar results also show effectiveness through the folate-conjugated human serum albumin RSV NPs (FA-HSA-RESNPs) *versus* RSV alone in improved bioavailability (Lian et al., 2019). Inhibition of the cell proliferation process in hepatic tumors was also evident in the RSV-conjugated form, compared to the RSV alone injected into the rats (Lian et al., 2019). Similar NPs to those shown in Wu’s and Lian’s studies may be potential carriers of RSV to enhance the compound’s anti-cancer effects in MM cells. Although the *in vitro* studies described in section 3.4 illustrate the beneficial potential of RSV alone, MM patients treated with micronized RSV in a phase II study experienced several adverse effects, exhibiting a poor safety profile for a 5g daily dose of RSV (Popat et al., 2013). The application of an NP carrier, as supported by the rat studies by Wu et al. and Lian et al., could be a potential solution for these adverse effects.

In particular, applying an NP carrier system to RSV and its derivatives increases the likelihood of delivering the preferred therapeutic outcome of the drug to the MM cells in the bone marrow. To deliver RSV and other natural compounds, more types of NPs can be applied to specifically target MM cells, such as the PEGylated micellar NPs with VLA-4-antagonist peptides (Kiziltepe et al., 2012). The VLA-4 antagonist peptide acts to prevent the adhesion of MM cells to fibronectin, which is important in addressing cell-adhesion-mediated drug resistance (Kiziltepe et al., 2012). Because of this targeted approach, RSV is more effectively delivered to the malignant cells, potentially allowing lower doses to be effective and minimizing adverse off-target effects associated with high doses.

### 4.5 Curcumin

To further improve the bioavailability and distribution of CUR to the designated target site within cancer cells, NP carrier systems have been applied to enhance the delivery of CUR and its combination therapy treatment with other anti-cancer drugs. Recent studies show the development of drug encapsulation of CUR with chitosan (CS) nanocarrier increases the protective layer of CUR with the addition of Fe_3_O_4_-shell Au and folic acid NPs (Fe@Au-CU-CS-FA NPs) (Al-Kinani et al., 2021). The Fe@Au-CU-CS-FA NPs exhibited a spherical uniform shape with high encapsulation efficiency of 82% (Al-Kinani et al., 2021). These specific NPs inhibited human lung adenocarcinoma A549 cell proliferation significantly compared to the NP and CUR treatments alone, but needed micromolar concentration (Al-Kinani et al., 2021). The safety and efficacy of this study support further research of these CUR-loaded NPs on other cancer cell lines, like MM cells. Establishing CUR’s stability and distribution allows for additional studies involving combinatorial anti-cancer drug treatments to enhance the cytotoxic effects of CUC in cancer cells. One study investigated CD44-targeting doxorubicin (DOX) loaded NPs with CUR-loaded selenium cell-receptor targeted NPs in an effort to reduce the development of potential drug resistance and further upregulate cell cycle arrest and apoptosis in proliferating cancer cells (Kumari et al., 2018). The two treatments exhibited a combinatorial effect by further reducing cell viability of human colorectal HCT 116 cells and increasing ROS levels (Kumari et al., 2018). DOX is a chemotherapeutic drug used to treat MM. Hence, these results may point to a potential combinational therapy of DOX or other anti-cancer treatments with CUR to be used for MM (Turner et al., 2016).

Other studies have expanded further into different formulations of NPs, including assimilating silver NPs with CUR and clay minerals to obtain a chitosan composite film (Li et al., 2022). Li et al. found these NPs to have enhanced antioxidant and antibacterial activity, and the application of composite film *versus* the chitosan films increased the tensile strength and elongation at break (Li et al., 2022). The results offer a potential CUR-loaded NP delivery option for MM cells as well. Another potential drug therapy is the synergistic compound of both CUR and RSV in solid NPs for the effect of inhibiting the proliferation of melanoma cells (Palliyage et al., 2021). The results demonstrated a potent synergistic inhibition of B16F10 and SK-MEL-28 melanoma cell proliferation with CUR-RSV solid NPs at a ratio of 3:1 (Palliyage et al., 2021). This offers a direction for future studies to investigate the combination of natural compounds for enhancing cell cytotoxicity in MM cells.

### 4.6 Gallic acid

Multiple studies have been performed to encapsulate GA into NP carriers to achieve a more controlled release of this compound within the body. One controlled-release study found that iron oxide-chitosan-gallic acid (FCG) nanocarriers showed a fast release rate at the beginning of the study when GA anions were released (Dorniani et al., 2012). This was followed by 70% release during the first 150 min and a slower release of 96.7% for the next 1,200 min of release, which is due to the exchange of GA in the nanocarrier core with the anions in the solution (Dorniani et al., 2012). The pH of the solution the nanocarrier was submerged in significantly impacted the release of GA, with the release rate being lower at pH 7.4 *versus* that at pH 4.8 (Dorniani et al., 2012). This study also showed that the nanocarrier increased the thermal stability of GA and resulted in enhancing cytotoxic effects *versus* GA alone, depending on dosage and cancer type (Dorniani et al., 2012). Another study examined a different NP formulation in the form of PLGA NPs coated with polysorbate 80 (PS80) or without coating, each containing GA (de Cristo Soares Alves et al., 2016). During the controlled-release assay *in vitro*, there was a sustained release of GA from the uncoated NPs, whereas the PS80-coated NPs decreased GA release (de Cristo Soares Alves et al., 2016). GA-loaded PLGA NPs demonstrated no hemolysis in normal erythrocytes at all analyzed concentrations, yet PS80 NPs containing GA were cytotoxic at higher concentrations. Additionally, PLGA uncoated NPs had significantly more antioxidant activity than PS80-coated NPs (de Cristo Soares Alves et al., 2016). These results highlight the importance of developing NPs that will provide the greatest benefit for maximizing the efficacy of the natural compound they carry. In another study, gum arabic-stabilized GA NPs were applied *in vitro*, using breast adenocarcinoma, hepatocellular, colorectal adenocarcinoma, and breast epithelial cancer cell lines (Hassani et al., 2020). The results showed that the gum arabic GA NPs further decreased the viability of the cancer cells vs. GA alone and exhibited low toxicity in normal cells (Hassani et al., 2020).

Further studies have incorporated the use of silver NPs (AgNPs) due to their biological features and effects on antioxidant and cytotoxic activity. A study found that AgNPs formulated with *Rhizophora apiculata* extract, in which GA is a component, had greater anti-inflammatory activity vs. the extract alone (Alsareii et al., 2022). Additionally, the GA-loaded NPs showed greater cytotoxic effects in the tested lung, skin, and oral cancer cells (Alsareii et al., 2022). Continued studies are being conducted in further detail to increase the stability of GA and potentially for use in MM, including injectable agarose hydrogels (Ying et al., 2022).

### 4.7 Evodiamine

EVO is a promising therapeutic compound for the treatment of various types of cancer. However, its ability to exert its beneficial effects depends on its ability to enter the cell and trigger apoptosis and cell cycle arrest. Also, the poor water solubility of EVO negatively impacts its dissolution and ability to permeate cell membranes and elicit its cytotoxic effects (Liu et al., 2017). This can be overcome *via* the delivery of evodiamine in nano-formulations, and it has been shown that the delivery of evodiamine using a nano-emulsion enhances its bioavailability and cancer cytotoxic effects (Liu et al., 2017).

A combination of EVO and DOX using a nano-drug delivery system of mitochondria-targeting micelles has shown antitumor activity using *in vitro* and *in vivo* murine breast cancer models (Tan et al., 2019). Another use of nanotechnology to optimize evodiamine’s bioavailability is the use of effervescent SiO_2_–drug–Na_2_CO_3_ composite NPs (ESNs). This drug delivery system is capable of self-disintegrating at the site of interest while avoiding systemic toxicity (Chen et al., 2021). While studies are limited for EVO in MM cells, this compound and its use in anti-cancer activity have become more relevant in newer studies and offer a potential adjuvant regimen to enhance malignant cell apoptosis.

### 4.8 Limitations

The variation of NP formulations to study anti-MM phytochemical efficacy across these studies limits direct comparison of anti-MM effects of phytochemical-carrying NPs covered in this review. Most MM studies focused on *in vitro* models, with several compounds or systems limited to only one or two *in vivo* investigations, such as GA and EVO. Additional *in vivo* analysis is necessary to further investigate potential treatments for downstream assessment in MM patients. Several discussed NPs in this review are FDA or EMA approved carriers for anti-cancer drugs, including NP-bound albumin, iron oxide NPs, and lipid-based NPs (Wicki et al., 2015; Rodríguez et al., 2022), highlighting their safety and efficacy in delivering anti-cancer drugs, while other systems require further *in vivo* study. However, even within the same NP type, it is vital to rigorously assess the safety of these carriers as variations can result in different patient outcomes. For instance, several studies prepared PLGA NPs yet ranged in their formulations and synthesis methods (Zhao et al., 2015; Qiao et al., 2016; Fonseca et al., 2021; de Cristo Soares Alves et al., 2016). Assaying different formulations is crucial for researchers to identify carriers with the greatest phytochemical delivery potential while minimizing adverse effects from the NPs themselves. The application of NPs has great potential in addressing the low bioavailability of natural compounds, with added strength in designs using ligands or materials that specifically target these malignant cells and/or their predominant bone marrow environment.

## 5 Natural compound-loaded nanoparticles for cancer and aging

Cancer is majorly related to aging, having several overlapping characteristics, including epigenetic changes, genomic instability, inflammation, and dysbiosis (López-Otín et al., 2023). Cancer is considered an aging-related disorder, as the incidence of common cancers increases around the age of 50 (Calcinotto et al., 2019). Aging-related injury is highly associated with oxidative stress (Bjørklund et al., 2022). Oxidative stress dysregulates several pathways, damages DNA, and causes chromosomal changes, all of which contribute to cancer and senescence, also a cause of aging (Kudryavtseva et al., 2016). Phytochemicals are often associated with their anti-aging benefits. Several MM-targeting phytochemicals, such as RSV, CUR, and GA, have anti-aging properties including anti-oxidative and anti-inflammatory effects (Bjørklund et al., 2022; Ojeaburu and Oriakhi, 2021). RSV has been shown to inhibit cAMP phosphodiesterases in normal cells (Park et al., 2012). Quercetin and tocotrienols can induce senescence in cancer cells while delaying senescence in normal cells (Malavolta et al., 2016). Xue et al. have discussed the beneficial application of phytochemical nano-emulsions to target a variety of aging-related disorders, including cancer (Xue et al., 2021). Studying phytochemicals within the context of increased targeting/bioavailability by NP carriers can have potentially positive effects on drug development for MM and other cancers, in addition to other aging-related disorders. As oncogenic stress can cause senescence through pathways such as DNA damage (Mallette and Ferbeyre, 2007), understanding the multitargeting potential of phytochemicals can be beneficial for supporting the function of normal cells while addressing the concerns of malignant cells.

## 6 Conclusion

**FIGURE 4:**
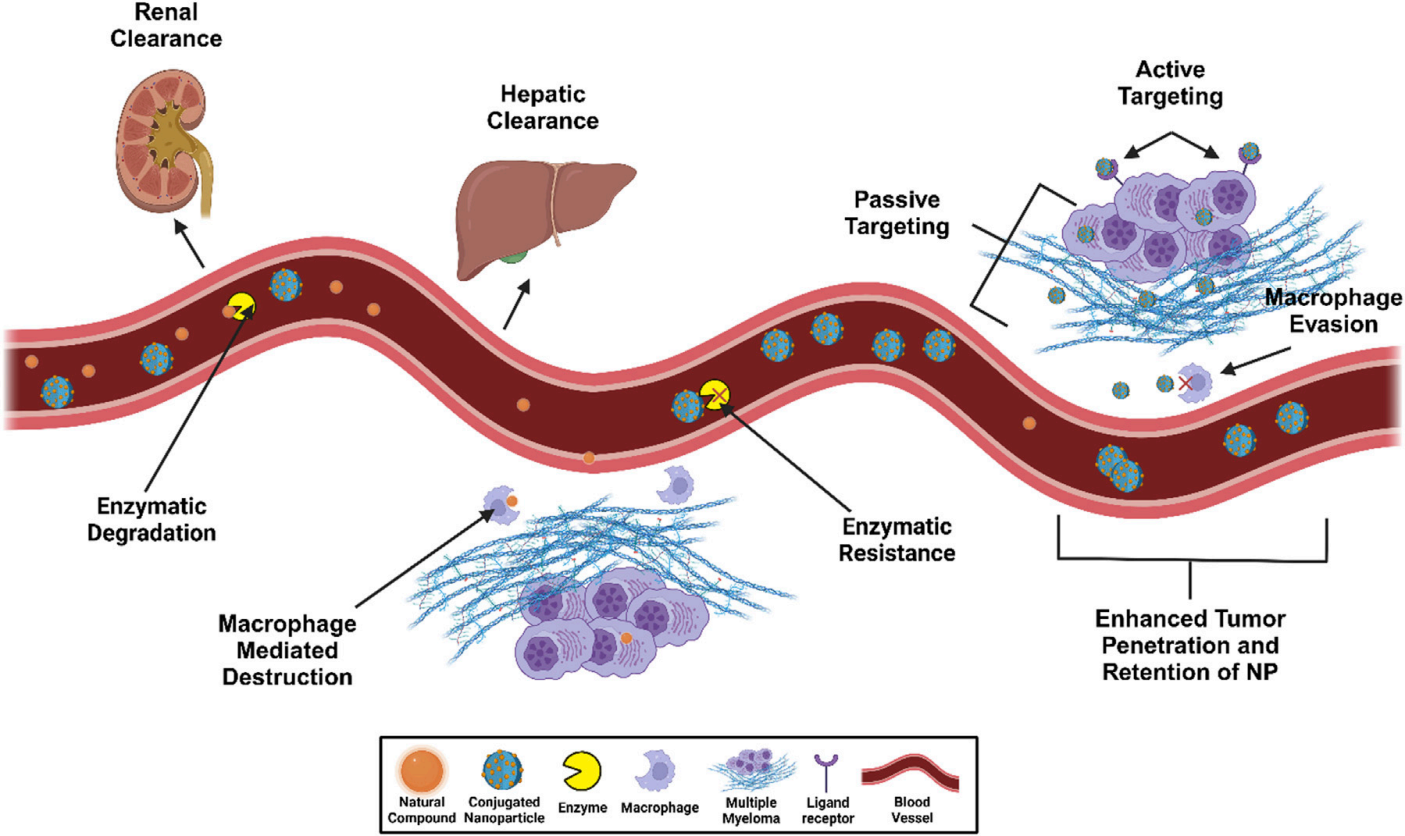
NP-enhanced targeted delivery against enzymatic degradation and macrophage-medicated destruction of natural compounds in multiple myeloma intervention. This figure was created through www.biorender.com (BioRender, Toronto, ON, Canada).

Ongoing research into MM treatment remains crucial due to the systemic adverse effects associated with current therapeutic options and the recurrent nature of this malignancy. Several plant-derived compounds discussed here have demonstrated promising anti-cancer properties, selectively targeting malignant cells while sparing normal cells. While the anti-MM effects of compounds, including RSV and CUR, have been extensively studied, compounds like GA and EVO require further analysis to identify MM mechanisms and subsequent investigation in *in vivo* models. Incorporating NP delivery systems has emerged as a transformative approach, significantly enhancing these natural compounds’ bioavailability, stability, and therapeutic efficacy, especially compared to the administration of the natural compounds alone, as illustrated in Figure 4. Although many NP formulations improve anti-MM phytochemical efficacy, the application of specific MM cell targeting or bone homing materials may be particularly beneficial in improving delivery to malignant cells and requiring lower phytochemical doses. Continuous advancements in NP design are further optimizing targeted delivery, improving treatment outcomes, and minimizing off-target effects. Beyond cancer treatment, plant-derived compounds offer substantial health benefits for both individuals with and without pathological conditions. The application of NP technology holds immense potential to amplify these benefits, paving the way for more effective and safer therapeutic interventions in oncology and age-related diseases.

## References

[B1] Al-Kinani M. A. Haider A. J. Al-Musawi S. (2021). Design, construction and characterization of intelligence polymer coated core–shell nanocarrier for curcumin drug encapsulation and delivery in lung cancer therapy purposes. J. Inorg. Organomet. Polym. Mater. 31, 70–79. 10.1007/s10904-020-01672-w

[B2] Allegra A. Speciale A. Molonia M. S. Guglielmo L. Musolino C. Ferlazzo G. (2018). Curcumin ameliorates the *in vitro* efficacy of carfilzomib in human multiple myeloma U266 cells targeting p53 and NF-κB pathways. Toxicol Vitro 47, 186–194. 10.1016/j.tiv.2017.12.001 29223572

[B3] Alsareii S. A. Manaa Alamri A. Alasmari M. Y. Bawahab M. A. Mahnashi M. H. Shaikh I. A. (2022). Synthesis and characterization of silver nanoparticles from *Rhizophora apiculata* and studies on their wound healing, antioxidant, anti-inflammatory, and cytotoxic activity. Molecules 27, 6306. 10.3390/molecules27196306 36234841 PMC9571849

[B4] Altayli E. Koru Ö. Öngörü Ö. İde T. Açikel C. Sarper M. (2015). An *in vitro* and *in vivo* investigation of the cytotoxic effects of caffeic acid (3,4-dihydroxycinnamic acid) phenethyl ester and bortezomib in multiple myeloma cells. Turk J. Med. Sci. 45, 38–46. 10.3906/sag-1401-127 25790528

[B5] Andreani T. Cheng R. Elbadri K. Ferro C. Menezes T. Dos Santos M. R. (2024). Natural compounds-based nanomedicines for cancer treatment: future directions and challenges. Drug Deliv. Transl. Res. 14, 2845–2916. 10.1007/s13346-024-01649-z 39003425 PMC11385056

[B6] Asci H. Ozmen O. Ellidag H. Y. Aydin B. Bas E. Yilmaz N. (2017). The impact of gallic acid on the methotrexate-induced kidney damage in rats. J. Food Drug Anal. 25, 890–897. 10.1016/j.jfda.2017.05.001 28987366 PMC9328864

[B7] Babar A. Babar M. Zubair H. Shahid A. Rafique S. Bano M. (2024). Selinexor for the treatment of patients with relapsed or refractory multiple myeloma. J. Oncol. Pharm. Pract. 30, 535–546. 10.1177/10781552241235902 38454813

[B8] Bai Q.-X. Zhang X.-Y. (2012). Curcumin enhances cytotoxic effects of bortezomib in human multiple myeloma H929 cells: potential roles of NF-κB/JNK. Int. J. Mol. Sci. 13, 4831–4838. 10.3390/ijms13044831 22606012 PMC3344248

[B9] Beauregard A.-P. Harquail J. Lassalle-Claux G. Belbraouet M. Jean-Francois J. Touaibia M. (2015). CAPE analogs induce growth arrest and apoptosis in breast cancer cells. Molecules 20, 12576–12589. 10.3390/molecules200712576 26184141 PMC6332101

[B10] Benboubker L. Dimopoulos M. A. Dispenzieri A. Catalano J. Belch A. R. Cavo M. (2014). Lenalidomide and dexamethasone in transplant-ineligible patients with myeloma. N. Engl. J. Med. 371, 906–917. 10.1056/NEJMoa1402551 25184863

[B11] Bergsagel P. L. Kuehl W. M. Zhan F. Sawyer J. Barlogie B. Shaughnessy J. JR (2005). Cyclin D dysregulation: an early and unifying pathogenic event in multiple myeloma. Blood 106, 296–303. 10.1182/blood-2005-01-0034 15755896 PMC1895118

[B12] Bharti A. C. Donato N. Aggarwal B. B. (2003). Curcumin (diferuloylmethane) inhibits constitutive and IL-6-inducible STAT3 phosphorylation in human multiple myeloma cells. J. Immunol. 171, 3863–3871. 10.4049/jimmunol.171.7.3863 14500688

[B13] Bianchi G. Munshi N. C. (2015). Pathogenesis beyond the cancer clone(s) in multiple myeloma. Blood 125, 3049–3058. 10.1182/blood-2014-11-568881 25838343 PMC4432002

[B14] Bianco P. Gehron Robey P. (2000). Marrow stromal stem cells. J. Clin. Invest. 105, 1663–1668. 10.1172/JCI10413 10862779 PMC378520

[B15] Bjørklund G. Shanaida M. Lysiuk R. Butnariu M. Peana M. Sarac I. (2022). Natural compounds and products from an anti-aging perspective. Molecules 27, 7084. 10.3390/molecules27207084 36296673 PMC9610014

[B16] Broyl A. Hose D. Lokhorst H. De Knegt Y. Peeters J. Jauch A. (2010). Gene expression profiling for molecular classification of multiple myeloma in newly diagnosed patients. Blood 116, 2543–2553. 10.1182/blood-2009-12-261032 20574050

[B17] Caillot M. Dakik H. Mazurier F. Sola B. (2021). Targeting reactive oxygen species metabolism to induce myeloma cell death. Cancers (Basel) 13, 2411. 10.3390/cancers13102411 34067602 PMC8156203

[B200] Calcinotto A. Kohli J. Zagato E. Pellegrini L. Demaria M. Alimonti A. (2019). Cellular Senescence: Aging, Cancer, and Injury. Physiological reviews 99(2), 1047–1047. 10.1152/physrev.00020.2018 30648461

[B18] Cappell K. M. Kochenderfer J. N. (2023). Long-term outcomes following CAR T cell therapy: what we know so far. Nat. Rev. Clin. Oncol. 20, 359–371. 10.1038/s41571-023-00754-1 37055515 PMC10100620

[B19] Carvalho F. S. Burgeiro A. Garcia R. Moreno A. J. Carvalho R. A. Oliveira P. J. (2014). Doxorubicin-induced cardiotoxicity: from bioenergetic failure and cell death to cardiomyopathy. Med. Res. Rev. 34, 106–135. 10.1002/med.21280 23494977

[B20] Chari A. Lonial S. Mark T. M. Krishnan A. Y. Stockerl-Goldstein K. E. Usmani S. Z. (2018). Results of an early access treatment protocol of daratumumab in United States patients with relapsed or refractory multiple myeloma. Cancer 124, 4342–4349. 10.1002/cncr.31706 30395359 PMC6587745

[B21] Charliński G. Tyczyńska A. Małecki B. Fornagiel S. Barchnicka A. Kołkowska A. (2021). Risk factors and causes of early mortality in patients with newly diagnosed multiple myeloma in a “real-world” study: experiences of the Polish Myeloma Group. Pol. Arch. Intern Med. 131, 527-534. 10.20452/pamw.15980 33908731

[B22] Chen M.-J. Chang W.-H. Lin C.-C. Liu C.-Y. Wang T.-E. Chu C.-H. (2008). Caffeic acid phenethyl ester induces apoptosis of human pancreatic cancer cells involving caspase and mitochondrial dysfunction. Pancreatology 8, 566–576. 10.1159/000159843 18824880

[B23] Chen T. Jiang Y. Wang C. Cai Z. Chen H. Zhu J. (2021). The pH-triggered drug release and simultaneous carrier decomposition of effervescent SiO_2_–drug–Na_2_CO_3_ composite nanoparticles: to improve the antitumor activity of hydrophobic drugs. RSC Adv. 11, 5335–5347. 10.1039/d0ra07896d 35423073 PMC8694630

[B24] Chen X. Wang J. Fu Z. Zhu B. Wang J. Guan S. (2017). Curcumin activates DNA repair pathway in bone marrow to improve carboplatin-induced myelosuppression. Sci. Rep. 7, 17724. 10.1038/s41598-017-16436-9 29255221 PMC5735145

[B25] Chiecchio L. Dagrada G. P. Ibrahim A. H. Dachs Cabanas E. Protheroe R. K. M. Stockley D. M. (2009). Timing of acquisition of deletion 13 in plasma cell dyscrasias is dependent on genetic context. Haematologica 94, 1708–1713. 10.3324/haematol.2009.011064 19996118 PMC2791926

[B26] Chng W. J. Gonzalez-Paz N. Price-Troska T. Jacobus S. Rajkumar S. V. Oken M. M. (2008). Clinical and biological significance of RAS mutations in multiple myeloma. Leukemia 22, 2280–2284. 10.1038/leu.2008.142 18528420 PMC3864109

[B27] Chng W. J. Huang G. F. Chung T. H. Ng S. B. Gonzalez-Paz N. Troska-Price T. (2011). Clinical and biological implications of MYC activation: a common difference between MGUS and newly diagnosed multiple myeloma. Leukemia 25, 1026–1035. 10.1038/leu.2011.53 21468039 PMC3432644

[B28] Cole D. C. Frishman W. H. (2018). Cardiovascular complications of proteasome inhibitors used in multiple myeloma. Cardiol. Rev. 26, 122–129. 10.1097/CRD.0000000000000183 29077585

[B29] Collins W. Lowen N. Blake D. J. (2019). Caffeic acid esters are effective bactericidal compounds against paenibacillus larvae by altering intracellular oxidant and antioxidant levels. Biomolecules 9, 312. 10.3390/biom9080312 31357646 PMC6722690

[B30] Costa L. J. Chhabra S. Medvedova E. Dholaria B. R. Schmidt T. M. Godby K. N. (2022). Daratumumab, carfilzomib, lenalidomide, and dexamethasone with minimal residual disease response-adapted therapy in newly diagnosed multiple myeloma. J. Clin. Oncol. 40, 2901–2912. 10.1200/JCO.21.01935 34898239

[B31] De Cristo Soares Alves A. Mainardes R. M. Khalil N. M. (2016). Nanoencapsulation of gallic acid and evaluation of its cytotoxicity and antioxidant activity. Mater Sci. Eng. C Mater Biol. Appl. 60, 126–134. 10.1016/j.msec.2015.11.014 26706515

[B32] Dei Cas M. Ghidoni R. (2019). Dietary curcumin: correlation between bioavailability and health potential. Nutrients 11, 2147. 10.3390/nu11092147 31500361 PMC6770259

[B33] Dimopoulos M. A. Hillengass J. Usmani S. Zamagni E. Lentzsch S. Davies F. E. (2015). Role of magnetic resonance imaging in the management of patients with multiple myeloma: a consensus statement. J. Clin. Oncol. 33, 657–664. 10.1200/JCO.2014.57.9961 25605835

[B34] Dorniani D. Kura A. U. Ahmad Z. Halim Shaari A. Hussein M. Z. Fakurazi S. (2012). Preparation of Fe_3_O_4_ magnetic nanoparticles coated with gallic acid for drug delivery. Int. J. Nanomedicine 5745, 5745. 10.2147/ijn.s35746 PMC3500033 23166439

[B35] Elwakeel S. H. B. Abdel Rahman A. (2021). Protective effect of gallic acid on cyclophosphamide-induced nephrotoxicity, oxidative stress, genotoxicity, and histopathological alterations in male albino rats. Egypt. Acad. J. Biol. Sci. B. Zoology 13, 225–244. 10.21608/eajbsz.2021.207518

[B36] Fang Q. Jiang S. Li C. (2019). Evodiamine selectively inhibits multiple myeloma cell growth by triggering activation of intrinsic apoptosis pathway. OncoTargets Ther. 12, 11383–11391. 10.2147/OTT.S235730 PMC6935306 31920329

[B37] Feng Q. Yao Q. Li B. Xie Y. Zhang H. Xu Z. (2020). Glycolysis is suppressed by DCZ0801-induced inactivation of the Akt/mTOR pathway in Multiple Myeloma. J. Cancer 11, 4907–4916. 10.7150/jca.45146 32626538 PMC7330679

[B38] Fonseca M. Macedo A. S. Lima S. A. C. Reis S. Soares R. Fonte P. (2021). Evaluation of the antitumour and antiproliferative effect of xanthohumol-loaded PLGA nanoparticles on melanoma. Materials 14, 6421. 10.3390/ma14216421 34771946 PMC8585140

[B39] Fonseca R. Debes-Marun C. S. Picken E. B. Dewald G. W. Bryant S. C. Winkler J. M. (2003). The recurrent IgH translocations are highly associated with nonhyperdiploid variant multiple myeloma. Blood 102, 2562–2567. 10.1182/blood-2003-02-0493 12805059

[B40] Friberg L. E. Henningsson A. Maas H. Nguyen L. Karlsson M. O. (2002). Model of chemotherapy-induced myelosuppression with parameter consistency across drugs. J. Clin. Oncol. 20, 4713–4721. 10.1200/JCO.2002.02.140 12488418

[B41] Gallo C. Dallaglio K. Bassani B. Rossi T. Rossello A. Noonan D. M. (2016). Hop derived flavonoid xanthohumol inhibits endothelial cell functions *via* AMPK activation. Oncotarget 7, 59917–59931. 10.18632/oncotarget.10990 27494895 PMC5312358

[B42] Ghobrial I. M. Rajkumar S. V. (2003). Management of thalidomide toxicity. J. Support Oncol. 1, 194–205. 15334875 PMC3134146

[B43] Giannoni E. Bianchini F. Masieri L. Serni S. Torre E. Calorini L. (2010). Reciprocal activation of prostate cancer cells and cancer-associated fibroblasts stimulates epithelial-mesenchymal transition and cancer stemness. Cancer Res. 70, 6945–6956. 10.1158/0008-5472.CAN-10-0785 20699369

[B44] Golombick T. Diamond T. H. Badmaev V. Manoharan A. Ramakrishna R. (2009). The potential role of curcumin in patients with monoclonal gammopathy of undefined significance--its effect on paraproteinemia and the urinary N-telopeptide of type I collagen bone turnover marker. Clin. Cancer Res. 15, 5917–5922. 10.1158/1078-0432.CCR-08-2217 19737963

[B45] Golombick T. Diamond T. H. Manoharan A. Ramakrishna R. (2012). Monoclonal gammopathy of undetermined significance, smoldering multiple myeloma, and curcumin: a randomized, double-blind placebo-controlled cross-over 4g study and an open-label 8g extension study. Am. J. Hematol. 87, 455–460. 10.1002/ajh.23159 22473809

[B46] Guo T. Fang X. Liu Y. Ruan Y. Hu Y. Wang X. (2022). Acute lung inflammation induced by zinc oxide nanoparticles: evolution and intervention via NRF2 activator. Food Chem. Toxicol. 162, 112898. 10.1016/j.fct.2022.112898 35247504

[B47] Han D. Ma G. Gao Y. Su Y. (2021). Curcumin synergistically enhances the cytotoxicity of arsenic trioxide in U266 cells by increasing arsenic uptake. Evidence-Based Complementary Altern. Med. 2021, 3083041–3083049. 10.1155/2021/3083041 PMC8526211 34675983

[B48] Haramizu S. Asano S. Butler D. C. Stanton D. A. Hajira A. Mohamed J. S. (2017). Dietary resveratrol confers apoptotic resistance to oxidative stress in myoblasts. J. Nutr. Biochem. 50, 103–115. 10.1016/j.jnutbio.2017.08.008 29053994 PMC5694367

[B49] Harikumar K. B. Kunnumakkara A. B. Ahn K. S. Anand P. Krishnan S. Guha S. (2009). Modification of the cysteine residues in IkappaBalpha kinase and NF-kappaB (p65) by xanthohumol leads to suppression of NF-kappaB-regulated gene products and potentiation of apoptosis in leukemia cells. Blood 113, 2003–2013. 10.1182/blood-2008-04-151944 18952893 PMC2947354

[B50] Harish V. Tewari D. Mohd S. Govindaiah P. Babu M. R. Kumar R. (2022). Quality by design based formulation of xanthohumol loaded solid lipid nanoparticles with improved bioavailability and anticancer effect against PC-3 cells. Pharmaceutics 14, 2403. 10.3390/pharmaceutics14112403 36365221 PMC9699314

[B51] Hassani A. Azarian M. M. S. Ibrahim W. N. Hussain S. A. (2020). Preparation, characterization and therapeutic properties of gum Arabic-stabilized gallic acid nanoparticles. Sci. Rep. 10, 17808. 10.1038/s41598-020-71175-8 33082415 PMC7576211

[B52] Hoshida Y. (2023). EGCG for hepatocellular carcinoma chemoprevention (CATCH-B). ClinicalTrials.gov identifier NCT06015022.

[B53] Huang J. Chen Z.-H. Ren C.-M. Wang D.-X. Yuan S.-X. Wu Q.-X. (2015). Antiproliferation effect of evodiamine in human colon cancer cells is associated with IGF-1/HIF-1α downregulation. Oncol. Rep. 34, 3203–3211. 10.3892/or.2015.4309 26503233

[B54] Ivanova B. Spiteller M. (2014). Evodiamine and rutaecarpine alkaloids as highly selective transient receptor potential vanilloid 1 agonists. Int. J. Biol. Macromol. 65, 314–324. 10.1016/j.ijbiomac.2014.01.059 24495556

[B55] Jiang C.-H. Sun T.-L. Xiang D.-X. Wei S.-S. Li W.-Q. (2018). Anticancer activity and mechanism of xanthohumol: a prenylated flavonoid from hops (*Humulus lupulus* L.). Front. Pharmacol. 9, 530. 10.3389/fphar.2018.00530 29872398 PMC5972274

[B56] Jin H. G. Wu G. Z. Wu G. H. Bao Y. G. (2018). Combining the mammalian target of rapamycin inhibitor, rapamycin, with resveratrol has a synergistic effect in multiple myeloma. Oncol. Lett. 15, 6257–6264. 10.3892/ol.2018.8178 29731844 PMC5920858

[B57] Kam A. Li K. M. Razmovski-Naumovski V. Nammi S. Chan K. Li G. Q. (2014). Gallic acid protects against endothelial injury by restoring the depletion of DNA methyltransferase 1 and inhibiting proteasome activities. Int. J. Cardiol. 171, 231–242. 10.1016/j.ijcard.2013.12.020 24388544

[B58] Kapetanovic I. M. Muzzio M. Huang Z. Thompson T. N. Mccormick D. L. (2011). Pharmacokinetics, oral bioavailability, and metabolic profile of resveratrol and its dimethylether analog, pterostilbene, in rats. Cancer Chemother. Pharmacol. 68, 593–601. 10.1007/s00280-010-1525-4 21116625 PMC3090701

[B59] Kastritis E. Gavriatopoulou M. Solia E. Theodorakakou F. Spiliopoulou V. Malandrakis P. (2023). Real world efficacy and toxicity of selinexor: importance of patient characteristics, dose intensity and post progression outcomes. Clin. Lymphoma Myeloma Leuk. 23, 844–849. 10.1016/j.clml.2023.07.013 37599164

[B60] Kazandjian D. Mailankody S. Korde N. Landgren O. (2014). Smoldering multiple myeloma: pathophysiologic insights, novel diagnostics, clinical risk models, and treatment strategies. Clin. Adv. Hematol. Oncol. 12, 578–587. 25654479

[B61] Kelly K. (2010). The history of medicine. Facts on File. New York City, NY.

[B62] Kenna H. A. Poon A. W. De Los Angeles C. P. Koran L. M. (2011). Psychiatric complications of treatment with corticosteroids: review with case report. Psychiatry Clin. Neurosci. 65, 549–560. 10.1111/j.1440-1819.2011.02260.x 22003987

[B63] Khaki Najafabadi I. Samuels J. Dansby-Sparks R. Rayalam S. Mody V. (2017). Magnetic drug delivery of xanthohumol to adipocytes using ultrasmall superparamagnetic iron oxide nanoparticles. Philadelphia, PA.

[B64] Khushnud T. Mousa S. A. (2013). Potential role of naturally derived polyphenols and their nanotechnology delivery in cancer. Mol. Biotechnol. 55, 78–86. 10.1007/s12033-012-9623-7 23371307

[B65] Killeen D. P. Watkins O. C. Sansom C. E. Andersen D. H. Gordon K. C. Perry N. B. (2017). Fast sampling, analyses and chemometrics for plant breeding: bitter acids, xanthohumol and terpenes in lupulin glands of hops (*Humulus lupulus*). Phytochem. Anal. 28, 50–57. 10.1002/pca.2642 27976466

[B66] Kim T. Y. Park J. Oh B. Min H. J. Jeong T.-S. Lee J. H. (2009). Natural polyphenols antagonize the antimyeloma activity of proteasome inhibitor bortezomib by direct chemical interaction. Br. J. Haematol. 146, 270–281. 10.1111/j.1365-2141.2009.07752.x 19500098

[B67] Kiziltepe T. Ashley J. D. Stefanick J. F. Qi Y. M. Alves N. J. Handlogten M. W. (2012). Rationally engineered nanoparticles target multiple myeloma cells, overcome cell-adhesion-mediated drug resistance, and show enhanced efficacy *in vivo* . Blood Cancer J. 2, e64. 10.1038/bcj.2012.10 22829966 PMC3346680

[B68] Kleczka A. Kubina R. Dzik R. Jasik K. Stojko J. Cholewa K. (2020). Caffeic acid phenethyl ester (CAPE) induced apoptosis in serous ovarian cancer OV7 cells by deregulation of BCL2/BAX genes. Molecules 25, 3514. 10.3390/molecules25153514 32752091 PMC7435968

[B69] Koosha S. Mohamed Z. Sinniah A. Ibrahim Z. A. Seyedan A. Alshawsh M. A. (2019). Antiproliferative and apoptotic activities of 8-prenylnaringenin against human colon cancer cells. Life Sci. 232, 116633. 10.1016/j.lfs.2019.116633 31278947

[B201] Kudryavtseva A. V. Krasnov G.S. Dmitriev A. A. Alekseev B. Y. Kardymon O. L. Sadritdinova A. L. (2016). Mitochondrial dysfunction and oxidative stress in aging and cancer. Oncotarget, 7(29), 44879–44905. 10.18632/oncotarget.9821 27270647 PMC5216692

[B70] Kuehl W. M. Bergsagel P. L. (2002). Multiple myeloma: evolving genetic events and host interactions. Nat. Rev. Cancer 2, 175–187. 10.1038/nrc746 11990854

[B71] Kumar S. K. Callander N. S. Adekola K. Anderson L. D. Baljevic M. Baz R. (2023). Multiple myeloma, version 2.2024, NCCN clinical practice Guidelines in oncology. J. Natl. Compr. Cancer Netw. 21, 1281–1301. 10.6004/jnccn.2023.0061 38081133

[B72] Kumari M. Purohit M. P. Patnaik S. Shukla Y. Kumar P. Gupta K. C. (2018). Curcumin loaded selenium nanoparticles synergize the anticancer potential of doxorubicin contained in self-assembled, cell receptor targeted nanoparticles. Eur. J. Pharm. Biopharm. 130, 185–199. 10.1016/j.ejpb.2018.06.030 29969665

[B73] Kuo H.-C. Kuo W.-H. Lee Y.-J. Lin W.-L. Chou F.-P. Tseng T.-H. (2006). Inhibitory effect of caffeic acid phenethyl ester on the growth of C6 glioma cells *in vitro* and *in vivo* . Cancer Lett. 234, 199–208. 10.1016/j.canlet.2005.03.046 15885897

[B74] Kyle R. A. Gertz M. A. Witzig T. E. Lust J. A. Lacy M. Q. Dispenzieri A. (2003). Review of 1027 patients with newly diagnosed multiple myeloma. Mayo Clin. Proc. Engl. 78, 21–33. 10.4065/78.1.21 12528874

[B75] López-Otín C. Pietrocola F. Roiz-Valle D. Galluzzi L. Kroemer G. (2023). Meta-hallmarks of aging and cancer. Cell Metab. 35, 12–35. 10.1016/j.cmet.2022.11.001 36599298

[B76] Lian B. Wu M. Feng Z. Deng Y. Zhong C. Zhao X. (2019). Folate-conjugated human serum albumin-encapsulated resveratrol nanoparticles: preparation, characterization, bioavailability and targeting of liver tumors. Artif. Cells, Nanomedicine, Biotechnol. 47, 154–165. 10.1080/21691401.2018.1548468 30686050

[B77] Liao C.-H. Pan S.-L. Guh J.-H. Chang Y.-L. Pai H.-C. Lin C.-H. (2005). Antitumor mechanism of evodiamine, a constituent from Chinese herb Evodiae fructus, in human multiple-drug resistant breast cancer NCI/ADR-RES cells *in vitro* and *in vivo* . Carcinogenesis 26, 968–975. 10.1093/carcin/bgi041 15705600

[B78] Liby K. T. Sporn M. B. (2012). Synthetic oleanane triterpenoids: multifunctional drugs with a broad range of applications for prevention and treatment of chronic disease. Pharmacol. Rev. 64, 972–1003. 10.1124/pr.111.004846 22966038 PMC3462991

[B79] Li Q. Yue Y. Chen L. Xu C. Wang Y. Du L. (2018). Resveratrol sensitizes carfilzomib-induced apoptosis via promoting oxidative stress in multiple myeloma cells. Front. Pharmacol. 9, 334. 10.3389/fphar.2018.00334 29867453 PMC5961230

[B80] Li S. Mu B. Zhang H. Kang Y. Wang A. (2022). Incorporation of silver nanoparticles/curcumin/clay minerals into chitosan film for enhancing mechanical properties, antioxidant and antibacterial activity. Int. J. Biol. Macromol. 223, 779–789. 10.1016/j.ijbiomac.2022.11.046 36370856

[B81] Liu G.-L. Han N.-Z. Liu S.-S. (2018). Caffeic acid phenethyl ester inhibits the progression of ovarian cancer by regulating NF-κB signaling. Biomed. Pharmacother. 99, 825–831. 10.1016/j.biopha.2018.01.129 29710481

[B82] Liu D. Ahmet A. Ward L. Krishnamoorthy P. Mandelcorn E. D. Leigh R. (2013). A practical guide to the monitoring and management of the complications of systemic corticosteroid therapy. Allergy Asthma Clin. Immunol. 9, 30. 10.1186/1710-1492-9-30 23947590 PMC3765115

[B83] Liu L. Sun X. Guo Y. Ge K. (2022a). Evodiamine induces ROS-Dependent cytotoxicity in human gastric cancer cells via TRPV1/Ca(2+) pathway. Chem. Biol. Interact. 351, 109756. 10.1016/j.cbi.2021.109756 34808100

[B84] Liu Q. Chen J. Qin Y. Jiang B. Zhang T. (2020). Zein/fucoidan-based composite nanoparticles for the encapsulation of pterostilbene: preparation, characterization, physicochemical stability, and formation mechanism. Int. J. Biol. Macromol. 158, 461–470. 10.1016/j.ijbiomac.2020.04.128 32348858

[B85] Liu S. Chen D. Yuan Y. Zhang X. Li Y. Yan S. (2017). Efficient intracellular delivery makes cancer cells sensitive to nanoemulsive chemodrugs. Oncotarget 8, 65042–65055. 10.18632/oncotarget.17536 29029410 PMC5630310

[B86] Liu S. Wang J. Chen J. Guan S. Zhang T. (2022b). Sustained delivery of gambogic acid from mesoporous rod-structure hydroxyapatite for efficient *in vitro* cancer therapy. Biomater. Adv. 137, 212821. 10.1016/j.bioadv.2022.212821 35929258

[B87] Lodé L. Eveillard M. Trichet V. Soussi T. Wuillème S. Richebourg S. (2010). Mutations in TP53 are exclusively associated with del(17p) in multiple myeloma. Haematologica 95, 1973–1976. 10.3324/haematol.2010.023697 20634494 PMC2966923

[B88] Lonial S. Dimopoulos M. Palumbo A. White D. Grosicki S. Spicka I. (2015). Elotuzumab therapy for relapsed or refractory multiple myeloma. N. Engl. J. Med. 373, 621–631. 10.1056/NEJMoa1505654 26035255

[B89] Magalhães P. J. Carvalho D. O. Cruz J. M. Guido L. F. Barros A. A. (2009). Fundamentals and health benefits of xanthohumol, a natural product derived from hops and beer. Nat. Prod. Commun. 4, 591–610. 10.1177/1934578x0900400501 19445313

[B90] Maiocchi S. Cartaya A. Thai S. Akerman A. Bahnson E. (2022). Antioxidant Response Activating nanoParticles (ARAPas) localize to atherosclerotic plaque and locally activate the Nrf2 pathway. Biomaterials Sci. 10, 1231–1247. 10.1039/d1bm01421h PMC9181183 35076645

[B91] Malavolta M. Pierpaoli E. Giacconi R. Costarelli L. Piacenza F. Basso A. (2016). Pleiotropic effects of tocotrienols and quercetin on cellular senescence: introducing the perspective of senolytic effects of phytochemicals. Curr. Drug Targets 17, 447–459. 10.2174/1389450116666150907105104 26343116

[B92] Mallette F. A. Ferbeyre G. (2007). The DNA damage signaling pathway connects oncogenic stress to cellular senescence. Cell Cycle 6, 1831–1836. 10.4161/cc.6.15.4516 17671427

[B93] Maneix L. Sweeney M. A. Lee S. Iakova P. Moree S. E. Sahin E. (2021). The mitochondrial protease LonP1 promotes proteasome inhibitor resistance in multiple myeloma. Cancers (Basel) 13, 843. 10.3390/cancers13040843 33671345 PMC7922145

[B94] Manier S. Sacco A. Leleu X. Ghobrial I. M. Roccaro A. M. (2012). Bone marrow microenvironment in multiple myeloma progression. J. Biomed. Biotechnol. 2012, 1–5. 10.1155/2012/157496 23093834 PMC3471001

[B95] Ma R. Yu D. Peng Y. Yi H. Wang Y. Cheng T. (2021). Resveratrol induces AMPK and mTOR signaling inhibition-mediated autophagy and apoptosis in multiple myeloma cells. Acta Biochim. Biophys. Sin. (Shanghai) 53, 775–783. 10.1093/abbs/gmab042 33891090

[B96] Marin E. H. Paek H. Li M. Ban Y. Karaga M. K. Shashidharamurthy R. (2019). Caffeic acid phenethyl ester exerts apoptotic and oxidative stress on human multiple myeloma cells. Investig. New Drugs 37, 837–848. 10.1007/s10637-018-0701-y 30465316

[B97] Matz E. L. Hsieh M. H. (2017). Review of advances in uroprotective agents for cyclophosphamide- and ifosfamide-induced hemorrhagic cystitis. Urology 100, 16–19. 10.1016/j.urology.2016.07.030 27566144

[B98] Mehta J. Singhal S. (2003). Hyperviscosity syndrome in plasma cell dyscrasias. Semin. Thromb. Hemost. 29, 467–471. 10.1055/s-2003-44554 14631546

[B99] Mei H. Xiang Y. Mei H. Fang B. Wang Q. Cao D. (2018). Pterostilbene inhibits nutrient metabolism and induces apoptosis through AMPK activation in multiple myeloma cells. Int. J. Mol. Med. 42, 2676–2688. 10.3892/ijmm.2018.3857 30226553 PMC6192759

[B100] Mena S. Rodríguez M. L. Ponsoda X. Estrela J. M. Jäättela M. Ortega A. L. (2012). Pterostilbene-induced tumor cytotoxicity: a lysosomal membrane permeabilization-dependent mechanism. PLoS One 7, e44524. 10.1371/journal.pone.0044524 22957077 PMC3434130

[B101] Merin N. M. Kelly K. R. (2014). Clinical use of proteasome inhibitors in the treatment of multiple myeloma. Pharm. (Basel) 8, 1–20. 10.3390/ph8010001 PMC4381198 25545164

[B102] Nagarajan S. Mohandas S. Ganesan K. Xu B. Ramkumar K. M. (2022). New insights into dietary pterostilbene: sources, metabolism, and health promotion effects. Molecules 27, 6316. 10.3390/molecules27196316 36234852 PMC9571692

[B103] Neelapu S. S. Tummala S. Kebriaei P. Wierda W. Gutierrez C. Locke F. L. (2018). Chimeric antigen receptor T-cell therapy - assessment and management of toxicities. Nat. Rev. Clin. Oncol. 15, 47–62. 10.1038/nrclinonc.2017.148 28925994 PMC6733403

[B104] Newman D. J. Cragg G. M. (2020). Natural products as sources of new drugs over the nearly four decades from 01/1981 to 09/2019. J. Nat. Prod. 83, 770–803. 10.1021/acs.jnatprod.9b01285 32162523

[B105] Niesvizky R. Naib T. Christos P. J. Jayabalan D. Furst J. R. Jalbrzikowski J. (2007). Lenalidomide-induced myelosuppression is associated with renal dysfunction: adverse events evaluation of treatment-naïve patients undergoing front-line lenalidomide and dexamethasone therapy. Br. J. Haematol. 138, 640–643. 10.1111/j.1365-2141.2007.06698.x 17686058

[B106] Ojeaburu S. I. Oriakhi K. (2021). Hepatoprotective, antioxidant and, anti-inflammatory potentials of gallic acid in carbon tetrachloride-induced hepatic damage in Wistar rats. Toxicol. Rep. 8, 177–185. 10.1016/j.toxrep.2021.01.001 33489777 PMC7806503

[B107] Pabla N. Dong Z. (2008). Cisplatin nephrotoxicity: mechanisms and renoprotective strategies. Kidney Int. 73, 994–1007. 10.1038/sj.ki.5002786 18272962

[B108] Palliyage G. H. Hussein N. Mimlitz M. Weeder C. Alnasser M. H. A. Singh S. (2021). Novel curcumin-resveratrol solid nanoparticles synergistically inhibit proliferation of melanoma cells. Pharm. Res. 38, 851–871. 10.1007/s11095-021-03043-7 33982225

[B109] Palumbo A. Hajek R. Delforge M. Kropff M. Petrucci M. T. Catalano J. (2012). Continuous lenalidomide treatment for newly diagnosed multiple myeloma. N. Engl. J. Med. 366, 1759–1769. 10.1056/NEJMoa1112704 22571200

[B110] Pantuck A. J. Leppert J. T. Zomorodian N. Aronson W. Hong J. Barnard R. J. (2006). Phase II study of pomegranate juice for men with rising prostate-specific antigen following surgery or radiation for prostate cancer. Clin. Cancer Res. 12, 4018–4026. 10.1158/1078-0432.CCR-05-2290 16818701

[B111] Parikh N. R. Mandal A. Bhatia D. Siveen K. S. Sethi G. Bishayee A. (2014). Oleanane triterpenoids in the prevention and therapy of breast cancer: current evidence and future perspectives. Phytochem. Rev. 13, 793–810. 10.1007/s11101-014-9337-5 25395898 PMC4225818

[B112] Park S.-J. Ahmad F. Philp A. Baar K. Williams T. Luo H. (2012). Resveratrol ameliorates aging-related metabolic phenotypes by inhibiting cAMP phosphodiesterases. Cell 148, 421–433. 10.1016/j.cell.2012.01.017 22304913 PMC3431801

[B113] Peñalva R. Morales J. González-Navarro C. Larrañeta E. Quincoces G. Peñuelas I. (2018). Increased oral bioavailability of resveratrol by its encapsulation in casein nanoparticles. Int. J. Mol. Sci. 19, 2816. 10.3390/ijms19092816 30231546 PMC6163610

[B114] Petronelli A. Pannitteri G. Testa U. (2009). Triterpenoids as new promising anticancer drugs. Anticancer Drugs 20, 880–892. 10.1097/CAD.0b013e328330fd90 19745720

[B115] Pojero F. Poma P. Spanò V. Montalbano A. Barraja P. Notarbartolo M. (2019). Targeting multiple myeloma with natural polyphenols, Eur. J. Med. Chem. Fr. 180, 465-485. 10.1016/j.ejmech.2019.07.041 31330448

[B116] Popat R. Plesner T. Davies F. Cook G. Cook M. Elliott P. (2013). A phase 2 study of SRT501 (resveratrol) with bortezomib for patients with relapsed and or refractory multiple myeloma. Br. J. Haematol. 160, 714–717. 10.1111/bjh.12154 23205612

[B117] Possemiers S. Heyerick A. Robbens V. De Keukeleire D. Verstraete W. (2005). Activation of proestrogens from hops (Humulus lupulus L.) by intestinal microbiota; conversion of isoxanthohumol into 8-prenylnaringenin. J. Agric. Food Chem. 53, 6281–6288. 10.1021/jf0509714 16076107

[B118] Qiao T. Jiang S. Song P. Song X. Liu Q. Wang L. (2016). Effect of blending HA-g-PLLA on xanthohumol-loaded PLGA fiber membrane. Colloids Surf. B Biointerfaces 146, 221–227. 10.1016/j.colsurfb.2016.06.011 27343844

[B119] Qi R. Wang S. Yu J. Lu T. Bi Z. Liu W. (2024). Enhanced precision therapy of multiple myeloma through engineered biomimetic nanoparticles with dual targeting. Engineering 36, 178–192. 10.1016/j.eng.2024.01.001

[B120] Rajkumar S. V. (2019). Multiple myeloma: every year a new standard? Hematol. Oncol. 37, 62–65. 10.1002/hon.2586 PMC6570407 31187526

[B121] Richard E. T. Morinaga K. Zheng Y. Sundberg O. Hokugo A. Hui K. (2021). Design and synthesis of cathepsin-K-activated osteoadsorptive fluorogenic sentinel (OFS) probes for detecting early osteoclastic bone resorption in a multiple myeloma mouse model. Bioconjug Chem. 32, 916–927. 10.1021/acs.bioconjchem.1c00036 33956423 PMC8137654

[B122] Richardson P. G. Beksac M. Spicka I. Mikhael J. (2020). Isatuximab for the treatment of relapsed/refractory multiple myeloma. Expert Opin. Biol. Ther. 20, 1395–1404. 10.1080/14712598.2021.1841747 33111607

[B123] Richardson P. G. Weller E. Lonial S. Jakubowiak A. J. Jagannath S. Raje N. S. (2010). Lenalidomide, bortezomib, and dexamethasone combination therapy in patients with newly diagnosed multiple myeloma. Blood 116, 679–686. 10.1182/blood-2010-02-268862 20385792 PMC3324254

[B124] Rodríguez F. Caruana P. De La Fuente N. Español P. Gámez M. Balart J. (2022). Nano-based approved pharmaceuticals for cancer treatment: present and future challenges. Biomolecules 12, 784. 10.3390/biom12060784 35740909 PMC9221343

[B125] Rogers L. J. John T. Park J. Tucker M. Ma H. Wu Y. (2020). Growth inhibition and apoptosis of human multiple myeloma cells induced by 2-cyano-3,12-dioxooleana-1,9-dien-28-oic acid derivatives. Anticancer Drugs 31, 806–818. 10.1097/CAD.0000000000000941 32304407

[B126] Rozic G. Paukov L. Jakubikova J. Ben-Shushan D. Duek A. Leiba A. (2016). The novel compound STK405759 is a microtubule-targeting agent with potent and selective cytotoxicity against multiple myeloma *in vitro* and *in vivo* . Oncotarget 7, 62572–62584. 10.18632/oncotarget.11539 27613836 PMC5308747

[B127] Samuels J. S. Shashidharamurthy R. Rayalam S. (2018). Novel anti-obesity effects of beer hops compound xanthohumol: role of AMPK signaling pathway. Nutr. Metabolism 15, 42. 10.1186/s12986-018-0277-8 PMC6003190 29946343

[B128] San-Miguel J. F. Paiva B. Gutiérrez N. C. (2013). New tools for diagnosis and monitoring of multiple myeloma. American Society of Clinical Oncology Educational Book, e313–e318. Philadelphia, PA. 10.14694/EdBook_AM.2013.33.e313 23714534

[B129] Santosa D. Suharti C. Riwanto I. Dharmana E. Pangarsa E. A. Setiawan B. (2022). Curcumin as adjuvant therapy to improve remission in myeloma patients: a pilot randomized clinical trial. Casp. J. Intern Med. 13, 375–384. 10.22088/cjim.13.2.9 PMC9301229 35919637

[B130] Schneider Y. Chabert P. Stutzmann J. Coelho D. Fougerousse A. Gossé F. (2003). Resveratrol analog (Z)-3,5,4'-trimethoxystilbene is a potent anti-mitotic drug inhibiting tubulin polymerization. Int. J. Cancer 107, 189–196. 10.1002/ijc.11344 12949793

[B131] Sell M. Lopes A. R. Escudeiro M. Esteves B. Monteiro A. R. Trindade T. (2023). Application of nanoparticles in cancer treatment: a concise review. Nanomater. (Basel) 13, 2887. 10.3390/nano13212887 PMC10650201 37947732

[B132] Siegel R. L. Giaquinto A. N. Jemal A. (2024). Cancer statistics, 2024. CA A Cancer J. Clin. 74, 12–49. 10.3322/caac.21820 38230766

[B133] Singh G. Pai R. S. Pandit V. (2014). *In vivo* pharmacokinetic applicability of a simple and validated HPLC method for orally administered trans-resveratrol loaded polymeric nanoparticles to rats. J. Pharm. Investigation 44, 69–78. 10.1007/s40005-013-0105-0

[B134] Sławińska-Brych A. Zdzisińska B. Czerwonka A. Mizerska-Kowalska M. Dmoszyńska-Graniczka M. Stepulak A. (2019). Xanthohumol exhibits anti-myeloma activity *in vitro* through inhibition of cell proliferation, induction of apoptosis via the ERK and JNK-dependent mechanism, and suppression of sIL-6R and VEGF production. Biochimica Biophysica Acta (BBA) - General Subj. 1863, 129408. 10.1016/j.bbagen.2019.08.001 31386885

[B135] Smith B. Li Y. Fields T. Tucker M. Staskiewicz A. Wong E. (2024). Tumor integrin targeted theranostic iron oxide nanoparticles for delivery of caffeic acid phenethyl ester: preparation, characterization, and anti-myeloma activities. Front. Pharmacol. 15, 1325196. 10.3389/fphar.2024.1325196 38510655 PMC10952826

[B136] Staskiewicz A. Wong E. Tucker M. Farhin R. Park J. Saade R. (2023). Cytotoxic and apoptotic effects of pinostilbene and bortezomib combination treatment on human multiple myeloma cells. Int. J. Mol. Sci. 24, 12590. 10.3390/ijms241612590 37628771 PMC10454535

[B137] Sun L. Liu H. Ye Y. Lei Y. Islam R. Tan S. (2023). Smart nanoparticles for cancer therapy. Signal Transduct. Target. Ther. 8, 418. 10.1038/s41392-023-01642-x 37919282 PMC10622502

[B138] Swami A. Reagan M. R. Basto P. Mishima Y. Kamaly N. Glavey S. (2014). Engineered nanomedicine for myeloma and bone microenvironment targeting, Proc. Natl. Acad. Sci. U. S. A. 111, 10287–10292. 10.1073/pnas.1401337111 24982170 PMC4104924

[B139] Takada Y. Kobayashi Y. Aggarwal B. B. (2005). Evodiamine abolishes constitutive and inducible NF-kappaB activation by inhibiting IkappaBalpha kinase activation, thereby suppressing NF-kappaB-regulated antiapoptotic and metastatic gene expression, up-regulating apoptosis, and inhibiting invasion. J. Biol. Chem. 280, 17203–17212. 10.1074/jbc.M500077200 15710601

[B140] Talib W. H. Alsayed A. R. Barakat M. Abu-Taha M. I. Mahmod A. I. (2021). Targeting drug chemo-resistance in cancer using natural products. Biomedicines 9, 1353. 10.3390/biomedicines9101353 34680470 PMC8533186

[B141] Tambuwala M. M. Khan M. N. Thompson P. Mccarron P. A. (2019). Albumin nano-encapsulation of caffeic acid phenethyl ester and piceatannol potentiated its ability to modulate HIF and NF-kB pathways and improves therapeutic outcome in experimental colitis. Drug Deliv. Transl. Res. 9, 14–24. 10.1007/s13346-018-00597-9 30430451 PMC6328632

[B142] Tan Q. Zhang J. (2016). Evodiamine and its role in chronic diseases. Adv. Exp. Med. Biol. 929, 315–328. 10.1007/978-3-319-41342-6_14 27771931

[B143] Tan X. Zhou Y. Shen L. Jia H. Tan X. (2019). A mitochondria-targeted delivery system of doxorubicin and evodiamine for the treatment of metastatic breast cancer. RSC Adv. 9, 37067–37078. 10.1039/c9ra07096f 35539080 PMC9075594

[B144] Tomizawa A. Kanno S.-I. Osanai Y. Goto A. Sato C. Yomogida S. (2013). Induction of apoptosis by a potent caffeic acid derivative, caffeic acid undecyl ester, is mediated by mitochondrial damage in NALM-6 human B cell leukemia cells. Oncol. Rep. 29, 425–429. 10.3892/or.2012.2163 23229564 PMC3583534

[B145] Traversi G. Fiore M. Percario Z. Degrassi F. Cozzi R. (2017). The resveratrol analogue trimethoxystilbene inhibits cancer cell growth by inducing multipolar cell mitosis. Mol. Carcinog. 56, 1117–1126. 10.1002/mc.22578 27739192

[B146] Traversi G. Staid D. S. Fiore M. Percario Z. Trisciuoglio D. Antonioletti R. (2019). A novel resveratrol derivative induces mitotic arrest, centrosome fragmentation and cancer cell death by inhibiting γ-tubulin. Cell Div. 14, 3. 10.1186/s13008-019-0046-8 31007707 PMC6457039

[B147] Tucker M. (2020). “Anti-myeloma effects of xanthohumol on human multiple myeloma cells *in vitro* via induction of apoptosis and activation of AMPK pathway,” in PCOM biomedical studies student scholarship. Philadelphia, PA.

[B148] Turner J. G. Dawson J. L. Grant S. Shain K. H. Dalton W. S. Dai Y. (2016). Treatment of acquired drug resistance in multiple myeloma by combination therapy with XPO1 and topoisomerase II inhibitors. J. Hematol. Oncol. 9, 73. 10.1186/s13045-016-0304-z 27557643 PMC4997728

[B149] Vadhan-Raj S. Weber D. M. Wang M. Giralt S. A. Thomas S. K. Alexanian R. (2007). Curcumin downregulates NF-kB and related genes in patients with multiple myeloma: results of a phase I/II study. Blood 110, 1177. 10.1182/blood.v110.11.1177.1177

[B150] Verma S. Singh A. Mishra A. (2013). Gallic acid: molecular rival of cancer. Environ. Toxicol. Pharmacol. 35, 473–485. 10.1016/j.etap.2013.02.011 23501608

[B151] Vijaya Padma V. Sowmya P. Arun Felix T. Baskaran R. Poornima P. (2011). Protective effect of gallic acid against lindane induced toxicity in experimental rats. Food Chem. Toxicol. 49, 991–998. 10.1016/j.fct.2011.01.005 21219962

[B152] Wang Y.-Y. Zhe H. Zhao R. (2014). Preclinical evidences toward the use of triterpenoid CDDO-Me for solid cancer prevention and treatment. Mol. Cancer 13, 30. 10.1186/1476-4598-13-30 24552536 PMC3940295

[B153] Wang J. Bhargava P. Yu Y. Sari A. N. Zhang H. Ishii N. (2020). Novel caffeic acid phenethyl ester-mortalin antibody nanoparticles offer enhanced selective cytotoxicity to cancer cells. Cancers (Basel) 12, 2370. 10.3390/cancers12092370 32825706 PMC7564736

[B154] Wicki A. Witzigmann D. Balasubramanian V. Huwyler J. (2015). Nanomedicine in cancer therapy: challenges, opportunities, and clinical applications. J. Control. Release 200, 138–157. 10.1016/j.jconrel.2014.12.030 25545217

[B155] Wong A. H.-H. Shin E. M. Tergaonkar V. Chng W.-J. (2020). Targeting NF-κB signaling for multiple myeloma. Cancers (Basel) 12, 2203. 10.3390/cancers12082203 32781681 PMC7463546

[B156] Wu M. Zhong C. Deng Y. Zhang Q. Zhang X. Zhao X. (2020). Resveratrol loaded glycyrrhizic acid-conjugated human serum albumin nanoparticles for tail vein injection II: pharmacokinetics, tissue distribution and bioavailability. Drug Deliv. 27, 81–90. 10.1080/10717544.2019.1704944 31858857 PMC6968672

[B157] Xiang D. Wang D. He Y. Xie J. Zhong Z. Li Z. (2006). Caffeic acid phenethyl ester induces growth arrest and apoptosis of colon cancer cells via the β-catenin/T-cell factor signaling. Anti-Cancer Drugs 17, 753–762. 10.1097/01.cad.0000224441.01082.bb 16926625

[B158] Xiao H. Xiao Q. Zhang K. Zuo X. Shrestha U. K. (2010). Reversal of multidrug resistance by curcumin through FA/BRCA pathway in multiple myeloma cell line MOLP-2/R. Ann. Hematol. 89, 399–404. 10.1007/s00277-009-0831-6 19756599

[B159] Xie B. Xu Z. Hu L. Chen G. Wei R. Yang G. (2016). Pterostilbene inhibits human multiple myeloma cells via ERK1/2 and JNK pathway *in vitro* and *in vivo* . Int. J. Mol. Sci. 17, 1927. 10.3390/ijms17111927 27869675 PMC5133923

[B160] Xue F. Li X. Qin L. Liu X. Li C. Adhikari B. (2021). Anti-aging properties of phytoconstituents and phyto-nanoemulsions and their application in managing aging-related diseases. Adv. Drug Deliv. Rev. 176, 113886. 10.1016/j.addr.2021.113886 34314783

[B161] Ying H. Wang H. Jiang G. Tang H. Li L. Zhang J. (2022). Injectable agarose hydrogels and doxorubicin-encapsulated iron-gallic acid nanoparticles for chemodynamic-photothermal synergistic therapy against osteosarcoma. Front. Chem. 10, 1045612. 10.3389/fchem.2022.1045612 36385986 PMC9663816

[B162] Yu H. Tu Y. Zhang C. Fan X. Wang X. Wang Z. (2010). Evodiamine as a novel antagonist of aryl hydrocarbon receptor. Biochem. Biophys. Res. Commun. 402, 94–98. 10.1016/j.bbrc.2010.09.122 20888792

[B163] Zeng Y. Du Q. Zhang Z. Ma J. Han L. Wang Y. (2020). Curcumin promotes cancer-associated fibroblasts apoptosis via ROS-mediated endoplasmic reticulum stress. Arch. Biochem. Biophys. 694, 108613. 10.1016/j.abb.2020.108613 33010228

[B164] Zhang K. Zhou Y. Xiao C. Zhao W. Wu H. Tang J. (2019). Application of hydroxyapatite nanoparticles in tumor-associated bone segmental defect. Sci. Adv. 5, eaax6946. 10.1126/sciadv.aax6946 31414050 PMC6677551

[B165] Zhao Y. Huo M. Xu Z. Wang Y. Huang L. (2015). Nanoparticle delivery of CDDO-Me remodels the tumor microenvironment and enhances vaccine therapy for melanoma. Biomaterials 68, 54–66. 10.1016/j.biomaterials.2015.07.053 26264646 PMC4551104

[B166] Żołnierczyk A. K. Mączka W. K. Grabarczyk M. Wińska K. Woźniak E. Anioł M. (2015). Isoxanthohumol--Biologically active hop flavonoid. Fitoterapia 103, 71–82. 10.1016/j.fitote.2015.03.007 25771121

